# Overcoming Docetaxel Resistance in Prostate Cancer by Targeting Cell Cycle Progression with Narciclasine-Based Compounds

**DOI:** 10.1021/acsomega.6c05272

**Published:** 2026-09-02

**Authors:** Ranyelison Silva Machado, Kaio S. Gomes, Augusto B. Farias, Natália Meneses Araújo, Giovanna Barbosa Chanes, Laura Maria Pinto Dias, Bianca Zaia Franco Ferreira, Carla Cristina Lopes, Ileana G. S. Rubio, Fernando Moreira Simabuco, Daniela G. G. Rando, Hugo Pequeno Monteiro, Adolfo Garcia Erustes, Soraya Soubhi Smaili, David Ryffel, David Sarlah, Giselle Cerchiaro, João Henrique G. Lago, Rodrigo Esaki Tamura

**Affiliations:** † Cancer Molecular Biology Laboratory, Federal University of São Paulo, São Paulo, São Paulo 04039-032, Brazil; ‡ Posgraduate Program of Chemical Biology, Institute of Environmental, Chemical and Pharmaceutical Sciences, Federal University of São Paulo, Diadema São Paulo 09972-270, Brazil; § Center for Natural and Human Sciences, 74362 Federal University of ABC, Santo Andre São Paulo 09210-180, Brazil; ∥ Department of Biochemistry, Federal University of São Paulo, São Paulo, São Paulo 04044-020, Brazil; ⊥ Postgraduate Program of Pathology, Postgraduate Program of Biotechnology, Department of Biological Sciences, Federal University of São Paulo, Diadema, São Paulo 09972-270, Brazil; # Department of Biochemistry, Center for Cellular and Molecular Therapy - CTCMol, Federal University of São Paulo, São Paulo, São Paulo 04039-032, Brazil; ∇ Pharmacology Department, Federal University of São Paulo, São Paulo, São Paulo 04044-020, Brazil; ○ Department of Chemistry, Wiess School of Natural Sciences, 124473 Rice University, Houston, Texas 770055, United States; ◆ Department of Biochemical-Pharmaceutical Technology, Faculty of Pharmacy, University of São Paulo, São Paulo, São Paulo 05508-000, Brazil

## Abstract

Development of resistance to taxane-based chemotherapy, specifically docetaxel (DTX), in advanced prostate cancer is frequent. Natural compounds, such as narciclasine, an alkaloid derived from plants, offer a potentially advantageous approach. Docetaxel-resistant prostate cancer cell lines (DU145RST and PC3RST) were established and treated with narciclasine (**1**) and its derivatives, narciclasine-3,4-acetonide (**1a**) and 7-deoxynarciclasine (**1b**), and evaluated for cytotoxicity, cell proliferation, cell cycle progression, clonogenicity, and 3D spheroid growth. *In silico* target prediction was undertaken employing PharmMapper, and the molecular pathways involved in resistance were confirmed by western blot. The resistant cell lines showed an increase in IC_50_ values above 6 nM in DU145RST and 4 nM in PC3RST of docetaxel; molecular alterations included increased mTOR and S6 signaling pathways. Narciclasine-based compounds decreased cell viability within both sensitive and resistant lines, interestingly displaying increased potency in the resistant sublines alongside minimal toxicity in nontumorigenic cell types. Cell cycle assessments unraveled G2/M arrest, particularly in the resistant model, a decline in clonogenic capacity, 3D spheroid fragmentation, and reduced viability. The *in silico* analysis identified CDK2, alongside cyclin A2, as targets, which certainly bolsters findings of cell cycle abrogation. Reduced concentrations of both CDK2 and cyclin A2 confirm the observed effects. Overall, the tested compounds **1**, **1a**, and **1b** exhibited potent and selective antitumor activity in docetaxel-resistant prostate cancer models by impairing proliferation and inducing cell cycle arrest. Therefore, these compounds represent promising candidates to overcome taxane resistance through modulation of cell cycle regulators such as CDK2 and cyclin A2.

## Graphical abstract

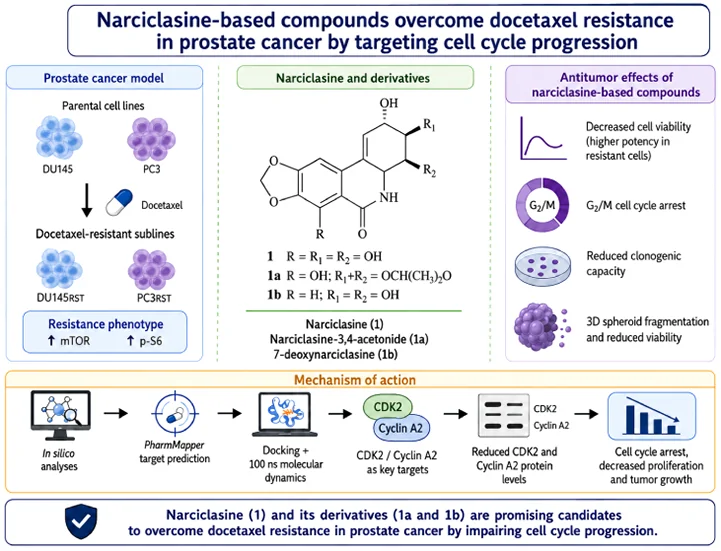

## 1 Introduction

Prostate cancer (PCa) remains a major global health burden. According to the latest GLOBOCAN estimates, PCa was the second most frequently diagnosed cancer and the fourth leading cause of cancer-related death among men worldwide in 2024, accounting for approximately 1.5 million new cases and 420,000 deaths.^1^ Although localized PCa can often be treated effectively, disease management becomes considerably more challenging following recurrence or metastatic dissemination.^2,3^ Through molecular evolution and treatment-driven selection, advanced tumors may progress despite androgen deprivation therapy (ADT), leading to castration-resistant prostate cancer (CRPC), which is defined by biochemical, radiographic, or clinical progression despite castrate serum testosterone levels.^3–5^ Depending on disease stage, risk classification, and prior treatment, therapeutic options include radical prostatectomy, radiotherapy, ADT, androgen receptor pathway inhibitors, chemotherapy, molecularly targeted therapies, and radioligand therapy.^5–8^

Chemotherapy remains an important component of treatment for advanced PCa, and docetaxel (DTX) is widely used because it improves overall survival in patients with advanced disease. DTX is a taxane that binds to and stabilizes microtubules, thereby disrupting microtubule dynamics, inducing G2/M cell cycle arrest, and ultimately promoting apoptosis.^9–12^ Despite its clinical efficacy, DTX can cause substantial adverse effects, including neutropenia, peripheral neuropathy, fatigue, and gastrointestinal symptoms such as vomiting and diarrhea, which may adversely affect patients’ quality of life.^13^ Moreover, the development of DTX resistance is associated with poorer clinical outcomes and shorter survival.^14^ Studies have reported a median overall survival of approximately 15 months among patients with DTX-resistant PCa, underscoring the need for effective therapeutic strategies for this population.^15–17^

The molecular mechanisms underlying resistance to chemotherapeutic agents are diverse and include the overexpression of drug efflux transporters, alterations in tubulin structure or isotype expression, dysregulation of DNA damage response and repair pathways, and activation of prosurvival and proliferative signaling cascades such as PI3K/AKT/mTOR.^18,19^ Additional mechanisms include epithelial–mesenchymal transition (EMT)^20–22^ and the dysregulation of tumor-suppressive microRNAs.^23^ Accordingly, there is a critical need to identify agents capable of killing resistant tumor cells or restoring their sensitivity to existing therapies.^24^ Owing to their substantial chemical diversity and bioactive potential, natural products, including taxanes and alkaloids, are valuable sources of candidate compounds for investigating tumor biology and mechanisms of treatment resistance.^25–28^ In particular, natural alkaloids with antimitotic or proapoptotic properties may exert antitumor activity either in combination with or independently of DTX, providing potential therapeutic strategies for DTX-resistant PCa.^25–28^

Narciclasine, an isocarbostyril alkaloid isolated from the bulbs of *Hymenocallis littoralis* (Amaryllidaceae), has demonstrated antitumor activity in cell lines derived from glioblastoma, melanoma, leukemia, and breast cancer. Its reported mechanisms of action include the inhibition of protein synthesis, induction of apoptosis, disruption of cytoskeletal organization, and modulation of stress-response pathways.^29–32^ However, its effects on PCa, particularly in DTX-resistant models, remain poorly understood. Induction of cell cycle arrest suppresses cell proliferation and may facilitate apoptotic cell death.^9–12^ Agents that promote cell cycle arrest may therefore enhance the antitumor effects of DTX.^9,10^ Because narciclasine inhibits cell proliferation, it represents a promising candidate for investigation in DTX-resistant PCa.^29^ In this study, we evaluated the antitumor activity of narciclasine (**1**) and its derivatives, narciclasine-3,4-acetonide (**1a**) and 7-deoxynarciclasine (**1b**) (Figure 1), in parental and DTX-resistant metastatic PCa cell lines.^33,34^ Our findings demonstrated that these compounds selectively inhibited tumor cell proliferation and exhibited greater activity in the resistant cell lines, supporting their potential to overcome DTX resistance in PCa. In addition, the compounds modulated the cell cycle regulators cyclin-dependent kinase 2 (CDK2) and cyclin A2 (CCNA2). We also performed molecular docking analyses to characterize the predicted interactions of these compounds with selected protein targets and examined how structural modifications of narciclasine and its derivatives influenced their predicted binding profiles.

**Figure 1:**
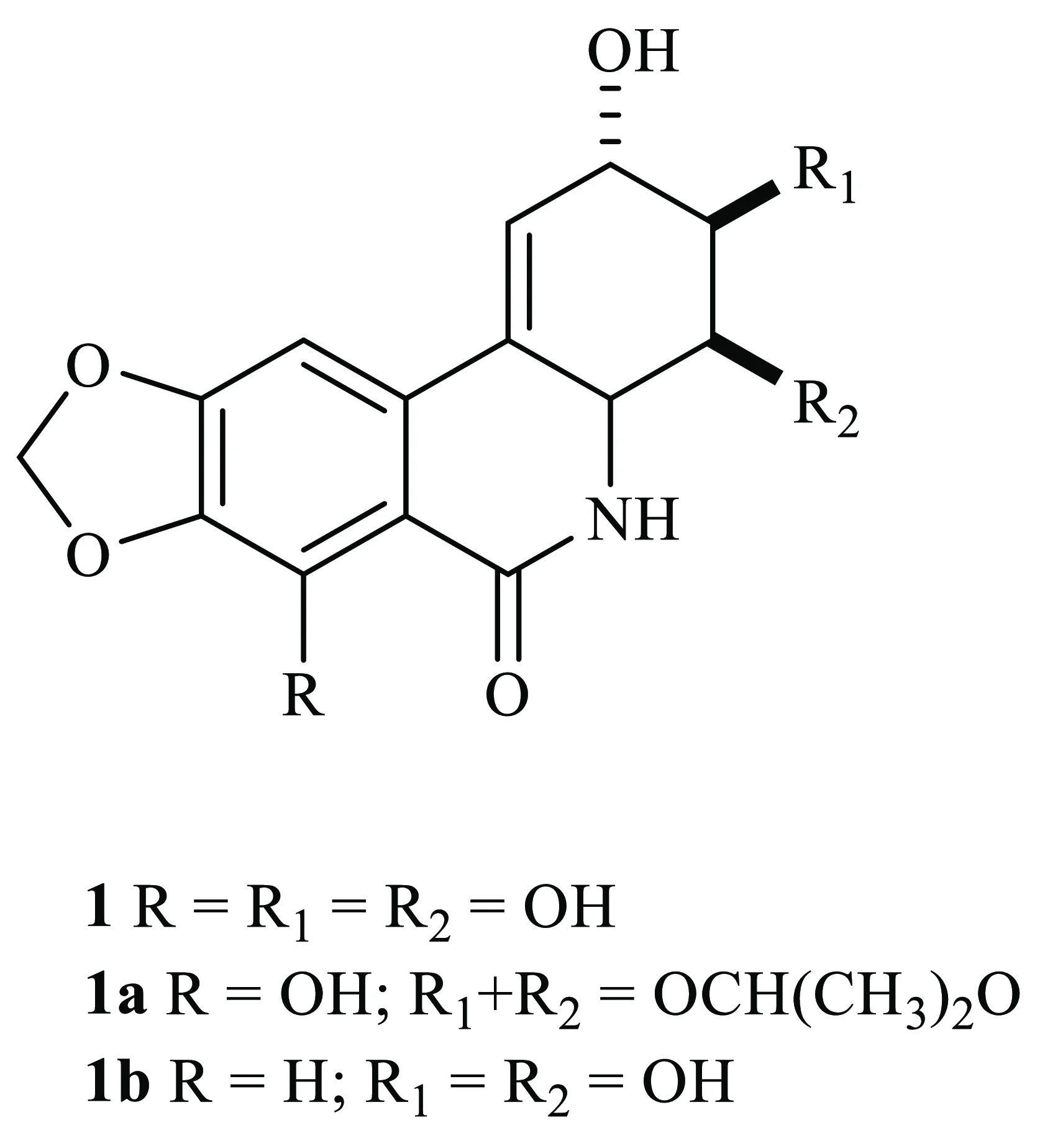
Structures of narciclasine (**1**) and its derivatives narciclasine-3,4-acetonide (**1a**) and 7-deoxynarciclasine (**1b**).

## 2 Materials and Methods

### 2.1 General

^1^H and ^13^C NMR spectra were recorded on a Varian INOVA 500 spectrometer (Palo Alto, CA, USA), operating at 500 and 125 MHz, respectively, using CD_3_OD and DMSO-*d*_6_ as solvents. Silica gel (230–400 mesh, Merck) was used for column chromatography (CC), and silica gel 60 PF_254_ (Macherey-Nagel) was used for analytical TLC. HPLC was performed using a Dionex Ultimate 3000 (Sunnyvale, CA, USA) chromatograph with a UVD-DAD-170 V detector.

### 2.2 Synthesis of Compounds **1**, **1a**, and **1b**

Details concerning the synthesis and structural characterization of compounds **1**, **1a**, and **1b** were previously described.^33,34^ As observed by HPLC analysis, these compounds were obtained with 100% purity.

### 2.3 Cell Lines and Culture Conditions

The human prostate cancer cell lines DU145 and PC3 and the nontumorigenic human umbilical vein endothelial cell (HUVEC) line were obtained from certified cell banks. The HUVEC cells were used as nontumor control to evaluate compound selectivity. In our laboratory, we developed DTX-resistant cell lines (DU145RST and PC3RST) by continuously increasing the dose of DTX in the parental cell lines, DU145 and PC3. Treatment began with a noncytotoxic dose of 0.5 nM DTX and increased by 0.25 nM every 15 days until reaching a maximum concentration of 5 nM, which is approximately the IC_50_ concentration of the parental cell lines. This concentration was not cytotoxic to the resistant cell lines but was lethal to the parental cell lines. All cells were maintained in Dulbecco’s modified Eagle medium (DMEM) supplemented with 10% fetal bovine serum (FBS) at 37 °C and 5% CO_2_.

### 2.4 IC_50_ Determination

IC_50_ values for docetaxel, compound **1**, and derivatives **1a** and **1b** were determined in DU145, PC3, DU145RST, PC3RST, and HUVEC cells. Cells were treated with a range of concentrations of each compound for 72 h. Viability was assessed by MTT assay, and IC_50_ values were calculated using nonlinear regression models.

### 2.5 Cell Viability Assay (MTT)

For MTT assays in monolayer cultures, 2 500 cells per well were seeded in 96-well plates and treated with increasing concentrations of each compound for 72 h. After treatment, an MTT solution (5 mg/mL in PBS) was added and incubated for 4 h. Formazan crystals were solubilized using lysis buffer (20% SDS in 50% DMF/2% acetic acid, pH adjusted to 4.7). Absorbance was measured at 570 nm by using a microplate reader.

### 2.6 Cell Count Assay

To assess cell proliferation over time, DU145, PC3, DU145RST, and PC3RST cells were seeded at 20,000 cells per well in 24-well plates. Cells were treated with the IC_50_ values of compounds **1**, **1a**, and **1b** and counted at 24, 48, and 72 h using Trypan Blue exclusion and a Neubauer hemocytometer.

### 2.7 Cell Cycle Analysis

Cells were plated at 200,000 cells per well in 6-well plates and treated with the IC_50_ values of compounds **1**, **1a,** and **1b** for 48 h. After treatment, cells were fixed in 70% ethanol and stored at −20 °C. Cells were incubated with a propidium iodide (PI) staining solution (50 μg/mL PI with RNase A in PBS) for 30 min at room temperature. DNA content was analyzed using a flow cytometer.

### 2.8 Clonogenic Assay

For colony formation assays, 500 cells per well were seeded in 6-well plates and treated with the IC_50_ value of compounds **1**, **1a**, and **1b** for 72 h. After drug removal, the cells were allowed to grow for 15 days. Colonies were fixed with methanol, stained with 0.5% crystal violet, and quantified by using ImageJ software.

### 2.9 3D Spheroid Viability Assay

Spheroids were generated using the hanging drop method. A cell mixture containing 7,500 cells in 120 μL of methylcellulose, with DMEM and FBS added to a final volume of 500 μL, was used to create 20 μL drops on the lid of 100 mm Petri dishes. After 48 h of aggregation, spheroids were transferred to ultralow attachment 96-well plates and treated for 72 h with 2 × IC_50_ concentrations of each tested compound. Cell viability was evaluated using PrestoBlue reagent (10 μL per well), and fluorescence was measured 6 h after reagent addition. Brightfield images were captured to assess spheroid morphology posttreatment.

### 2.10 Protein Expression Analysis: Western Blotting

Protein levels were assessed by western blot analysis. Membranes were incubated with the following primary antibodies: phospho-mTOR (p-mTOR) (1:1,000; Cell Signaling Technology, no. 2971), total mTOR (1:1,000; Cell Signaling Technology, no. 2983), phospho-S6K1 (p-S6K1) (1:1,000; Cell Signaling Technology, no. 9234), total S6K1 (1:1,000; Cell Signaling Technology, no. 2708), phospho-S6 (p-S6) (1:1,000; Cell Signaling Technology, no. 4858), total S6 (1:1,000; Cell Signaling Technology, no. 2217), CDK2 (1:1,000; Cell Signaling Technology, no. 18048S), Cyclin A (1:500; Santa Cruz Biotechnology, no. sc-271645), and GAPDH (1:10,000; Cell Signaling Technology, no. 2118), used as a loading control. After washing, membranes were incubated with HRP-conjugated secondary antibodies: antirabbit IgG-HRP (1:5,000; Cell Signaling Technology, no. 7074) or antimouse IgG-HRP (1:5,000; Cell Signaling Technology, no. 7076), depending on the host species of the primary antibody. Signal detection was performed using enhanced chemiluminescence (SuperSignal West Pico PLUS, Thermo Fisher Scientific, no. 34577). Protein bands were visualized using an ImageQuant LAS 4000 imaging system (GE Healthcare, USA). Quantification analysis was performed with ImageJ software (NIH, USA), and signal intensities were normalized to GAPDH. All western blotting experiments were independently repeated at least three times. Fold-change values were calculated relative to the loading controls, ensuring reproducibility and consistency across biological replicates.

### 2.11 Target Prediction Using Reverse Pharmacophore Mapping

The molecular targets of compounds **1**, **1a**, and **1b** were predicted using reverse pharmacophore mapping via the *PharmMapper* server (http://www.lilab-ecust.cn/pharmmapper/), developed by the Key Laboratory of Systems Biology, Shanghai Institutes for Biological Sciences, Chinese Academy of Sciences.

#### 2.11.1 Molecular Preparation

The structures of compounds **1** and **1b** were retrieved from *PubChem* (https://pubchem.ncbi.nlm.nih.gov), while the structure of compound **1a** was generated from its *SMILES notation* using *Avogadro* (version 1.2.0; https://avogadro.cc). All structures were subjected to geometry optimization using *Gaussian 09W* (Gaussian, Inc., Wallingford, CT, USA). Single-point energy calculations were then performed using the Hartree–Fock (HF) method with the 6-31G basis set. Partial atomic charges were recalculated using the *ChelpG* (Charges from Electrostatic Potentials using a Grid-based method) algorithm. The resulting files were saved in mol2 format using *GaussView 5.0* (Gaussian, Inc.) and were used as input to the *PharmMapper* platform.

#### 2.11.2 PharmMapper Settings

*PharmMapper* was configured to generate up to 300 low-energy conformers for each compound using its built-in conformational sampling algorithm. The screening was performed using the Human Protein Targets library (https://www.rcsb.org). For each compound, the top 100 targets were ranked according to the *Fit Score, Normalized Fit Score, and Z*′*-score.*

#### 2.11.3 Definition and Interpretation of the *Z*′-Score

The *Z*′-score is a standardized score that quantifies how well the compound’s pharmacophore matches a target relative to the mean and standard deviation of scores from the entire database. It is calculated as

$$Z^{\prime }=\frac{\mathrm{Fitscore}-\mu }{\sigma }$$

where μ is the mean fit score, and σ is the standard deviation across all targets in the library. Higher *Z*′-scores indicate better-than-average matches and are considered statistically significant. Typically, *Z*′-scores ≥ 2.0 are interpreted as reliable predictions, suggesting strong potential for interaction between the compound and the predicted protein target.^35^

### 2.12 Molecular Docking

Molecular docking simulations were performed using GNINA.^36^ Protein structures were prepared using ChimeraX 1.8 ^37^ by removing solvent molecules and nonessential ions. Polar hydrogen atoms and Gasteiger charges were assigned using AutoDock Tools.^38^ Docking calculations were conducted with an exhaustiveness of 32, 25 independent docking runs were performed, and the resulting poses were clustered using an RMSD tolerance of 2.0 Å. Binding modes were ranked according to predicted binding free energies and CNN scores, and the most favorable poses were selected for subsequent molecular dynamics simulations.

### 2.13 Molecular Dynamics

Molecular dynamics (MD) simulations were conducted using GROMACS 2024.3 with the CHARMM27 all-atom force field and the TIP3P explicit water model.^39^ Ligand parameters were generated using the SwissParam server.^40^ The protein–ligand complex was solvated in a triclinic simulation box with periodic boundary conditions, maintaining a minimum distance of 1.0 nm between the solute and the box edges. Na^+^ and Cl^–^ were added to neutralize the system and achieve a physiological ionic strength of 0.1 M. Energy minimization was performed using the steepest descent algorithm for 50,000 steps until convergence below 1,000 kJ/mol/nm. Equilibration was conducted in two stages with position restraints applied to heavy atoms: a 1.1 ns NVT phase at 300 K using the V-rescale thermostat, followed by a 100 ps NPT phase at 1.0 bar using the Berendsen barostat. The production MD simulation was performed for 100 ns in the NPT ensemble without restraints, using a 2 fs time step and the leapfrog integration algorithm. Long-range electrostatic interactions were treated using the particle mesh Ewald method with a cutoff of 1.2 nm, and all covalent bonds involving hydrogen atoms were constrained using the LINCS algorithm. Posttrajectory analyses, including RMSD, RMSF, and radius of gyration, were performed using standard GROMACS tools.

### 2.14 Cell Death Analysis

PC3, PC3RST, DU145, and DU145RST cells were seeded in 6-well plates at a density of 2 × 10^5^ cells per well. After 24 h, the cells were treated with vehicle control (DMSO), docetaxel (DTX) at the previously determined IC_50_ for each cell line, compound **1** at 0.05 μM for PC3 and PC3RST cells or 0.025 μM for DU145 and DU145RST cells, compound **1a** at 10 μM, or compound **1b** at 3 μM. After 72 h of treatment, both adherent and floating cells were collected. Aliquots containing 1 × 10^5^ cells were washed and resuspended in 1× binding buffer (10 mM HEPES, 140 mM NaCl, and 2.5 mM CaCl_2_, pH 7.4) and stained with 5 μL of Annexin V-FITC conjugate from the eBioscience Annexin V-FITC Apoptosis Detection Kit (Thermo Fisher Scientific, #BMS500FI-300) and 5 μL of ready-to-use eBioscience 7-AAD Viability Staining Solution (Thermo Fisher Scientific, #00-6993-50) per sample. The cell suspensions were incubated for 30 min at room temperature in the dark. Apoptotic and necrotic cell populations were subsequently quantified according to their Annexin V-FITC/7-AAD staining profiles using a BD LSRFortessa Cell Analyzer (BD Biosciences). All experiments were performed with a minimum of three independent biological replicates.

### 2.15 Wound-Healing Assay

Cell migration was evaluated using a wound-healing assay. PC3 and PC3RST cells were seeded in 48-well plates at a density of 5 × 10^4^ cells per well, whereas DU145 and DU145RST cells were seeded at 7.5 × 10^4^ cells per well. After 24 h, the cells were exposed to 1 μM mitomycin C for 2 h to minimize the contribution of cell proliferation to wound closure. Mitomycin C was prepared as a 1 mg/mL stock solution (Cayman Chemical Company, #11435) and diluted in culture medium to the final working concentration. Subsequently, a linear wound was generated in each cell monolayer using a sterile 200 μL pipet tip. The wells were gently washed to remove detached cells, and fresh culture medium containing vehicle control (DMSO), DTX at the previously determined IC_50_ for each cell line, compound **1** at 0.05 μM for PC3 and PC3RST cells or 0.025 μM for DU145 and DU145RST cells, compound **1a** at 10 μM, or compound **1b** at 3 μM was added. Images of the wounded areas were acquired at 24, 48, and 72 h using an EVOS M7000 Imaging System (Thermo Fisher Scientific) equipped with a 4× objective.

## 3 Results

### 3.1 Establishment and Characterization of Docetaxel-Resistant Prostate Cancer Cell Lines

After 72 h of treatment with increasing doses of docetaxel, the viability of DU145, PC3, DU145RST, and PC3RST cells was assessed using the MTT reagent. As shown in Figure 2A,B, the parental cell lines DU145 and PC3 exhibited lower viability. Viability was reduced to approximately 40–50% at concentrations between 2.5 and 5 nM DTX, and dropped to less than 20% at concentrations ≥10 nM, demonstrating a dose-dependent decrease. In contrast, the DTX-resistant cell lines DU145RST and PC3RST were less affected at the same concentrations. The IC_50_ values increased in the resistant cell lines. While the IC_50_ values of the parental cell lines DU145 and PC3 were close to 3 nM, the IC_50_ values of the resistant cell lines DU145RST and PC3RST were between 4.5 and 6 nM (Figure 2C and D). This is equivalent to twice the concentration of docetaxel needed to reach the IC_50_, indicating a reduction in the drug’s effectiveness.

**Figure 2:**
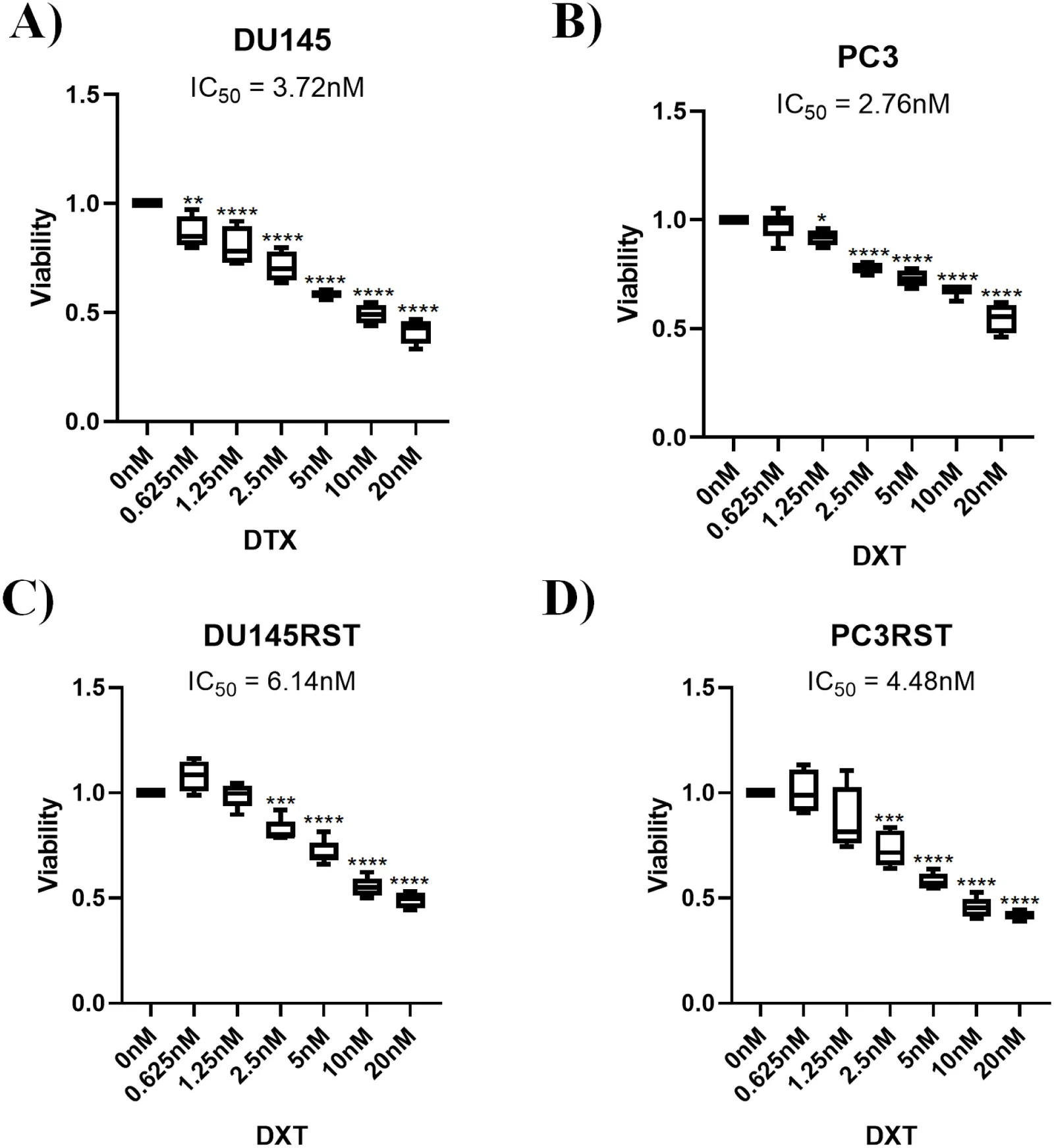
Establishment of docetaxel-resistant DU145RST and PC3RST prostate cancer sublines. Cell viability curves of parental (A) DU145 and (B) PC3 cells and their resistant counterparts, (C) DU145RST and (D) PC3RST, after 72 h of exposure to increasing concentrations of docetaxel, determined by MTT assay. Data are expressed as relative values of viable cells relative to DMSO-treated controls (mean ± SD, *n* = 3). IC_50_ values for docetaxel in parental and resistant cell lines, calculated from nonlinear regression of the dose–response curves. Representative graphs comparing mean IC_50_ values between parental and resistant lines. Statistical significance vs control: *p < 0.05, **p < 0.01, ***p < 0.001, ****p < 0.0001.

To better assess the molecular characteristics associated with the resistant phenotype, a western blot analysis was performed on key proteins involved in proliferation and survival pathways (Figure 3A). DU145RST and PC3RST cells expressed higher levels of total mTOR and S6K1 than did their parental counterparts. Phospho-mTOR levels increased slightly in the resistant cells. Despite the increase in total S6K1 levels, phospho-S6K1 levels decreased compared with parental cells. In contrast, phospho-S6 levels were clearly increased in both resistant sublines, accompanied by an increase in total S6. Overall, DU145RST and PC3RST cells exhibited higher total mTOR and S6K1 levels, reduced S6K1 phosphorylation, and strongly elevated levels of total and phosphorylated S6. The increase in mTOR and S6 expression in resistant cells indicates activation of the PI3K/AKT/mTOR cascade, an essential survival pathway in DTX resistance.^41–43^

**Figure 3:**
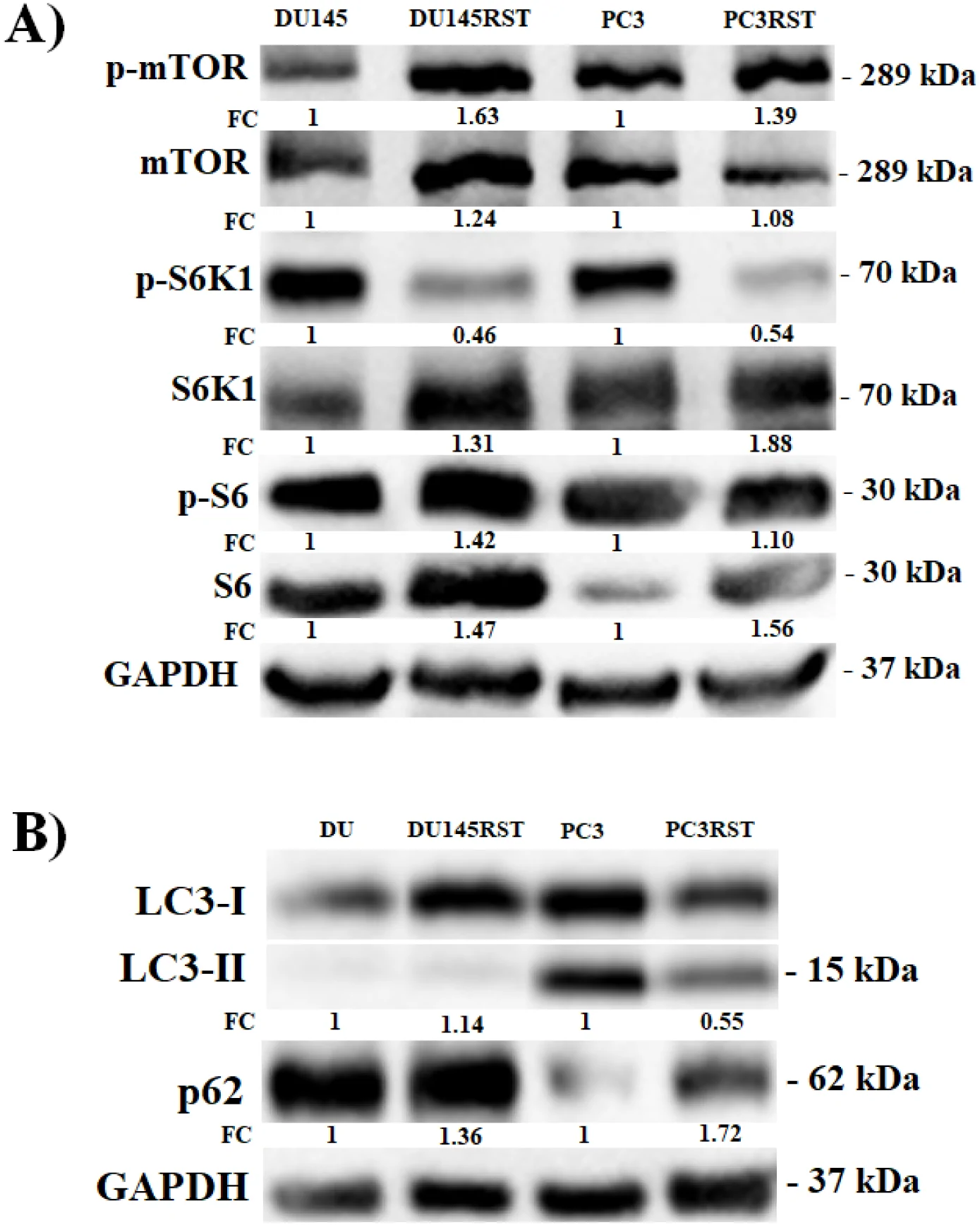
Characterization of docetaxel-resistant prostate cancer through protein expression profiles associated with proliferative signaling and autophagy. (A) Western blot analysis of total mTOR, phospho-mTOR, total S6K1, phospho-S6K1, total S6, and phospho-S6 in DU145, PC3, DU145RST, and PC3RST cells. (B) Western blot analysis of autophagy-related markers LC3B-I/II and p62. FC = fold-change. Representative cropped blots and quantifications are shown. Raw and uncropped blot images are provided in Supplementary Figure S1.

Autophagy-related proteins revealed differences among the cell lines (Figure 3B). DU145 and DU145RST cells had similar levels of LC3-II, whereas PC3RST cells had lower levels than the parental PC3 cells. The resistant cell lines showed an increased level of p62 expression. These findings confirm that DU145RST and PC3RST cells have distinct molecular and functional profiles that differentiate them from those of their parental cell lines. The raw and uncropped western blot images corresponding to Figure 3 are provided in Supplementary Figure S1.

### 3.2 Selective and Potentiated Cytotoxicity of Compounds **1**, **1a**, and **1b** in Docetaxel-Resistant Prostate Cancer

Figure 4 shows the dose–response graphs, which demonstrate that compounds **1**, **1a**, and **1b** significantly reduced viability in all tested cell lines. Table 1 shows that compound **1** demonstrated the highest cytotoxic potential in all prostate cancer cell lines. A comparison of the cell lines revealed that compound **1** was slightly more potent in the PC3RST cell line (IC_50_: 30.05 nM) than in the parental cell line (IC_50_: 53.40 nM). However, compound **1b** exhibited greater activity in resistant cells; it had a lower IC_50_ in DU145RST (IC_50_: 1.58 μM) than in DU145 (IC_50_: 2.59 μM). Compound **1a** demonstrated lower potency but still showed increased activity in resistant strains, particularly in PC3RST (IC_50_: 4.24 μM) compared to PC3 (IC_50_: 11.48 μM). In contrast, nontumorigenic HUVEC cells were less affected, particularly at concentrations that were effective in tumor cells. This highlights a favorable selectivity window.

**Table 1:** IC_50_ Values of Compounds **1**, **1a**, and **1b** in Prostate Cancer and Endothelial Cells

	**IC**_**50**_		
Cells lines	1(nM)	1a (μM)	1b (μM)
DU145	25.60	7.824	2.586
DU145RST	25.10	7.174	1.579
PC3	53.40	11.48	3.44
PC3RST	30.05	4.241	3.648
HUVEC	51.24	11.69	4.208

**Figure 4:**
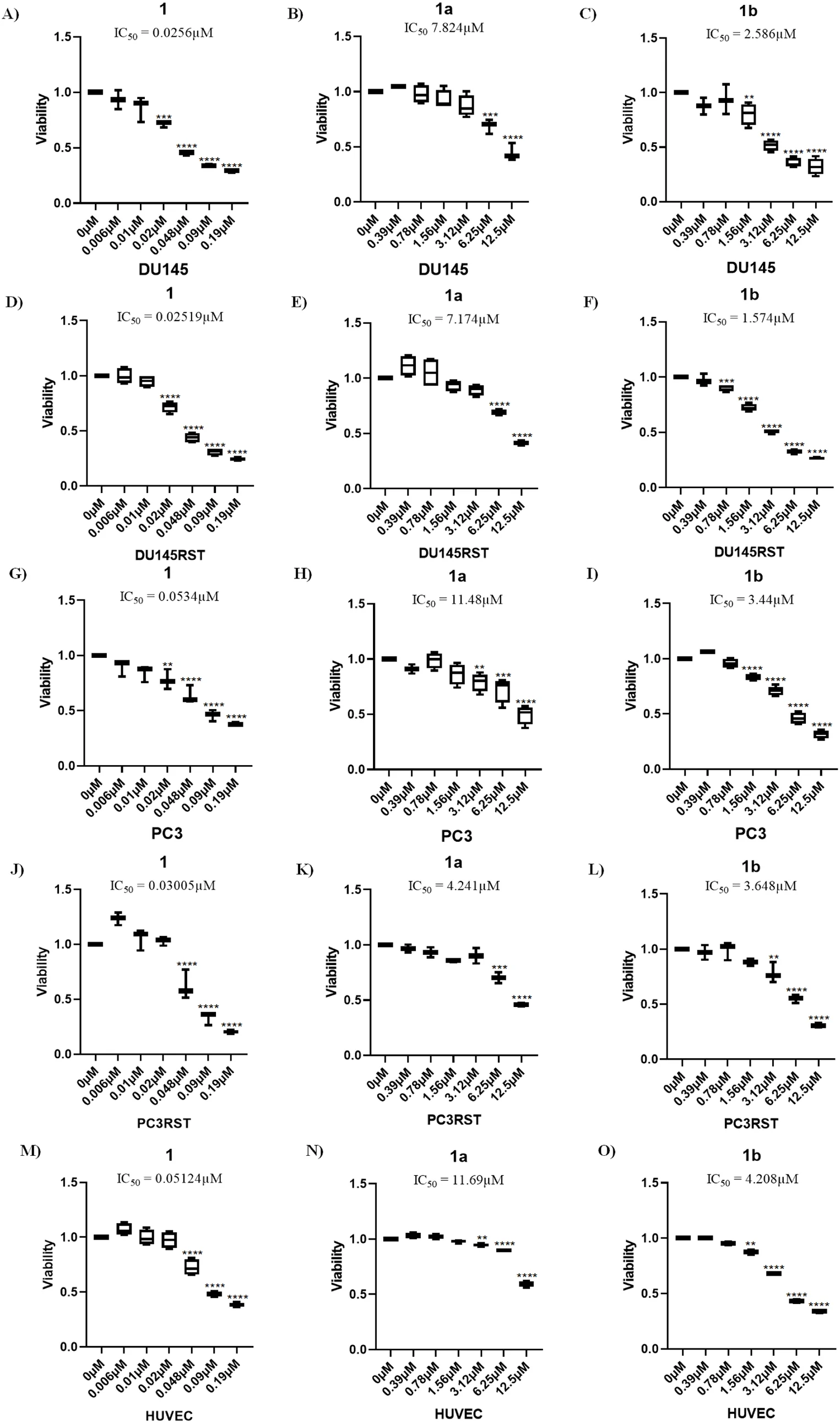
Dose–response curves of prostate cancer and HUVEC cells treated with compounds **1, 1a**, and **1b**. Cell viability was assessed using the MTT assay after 72 h of treatment with increasing concentrations of each compound. The panels represent (A) DU145 cells treated with compound **1**; (B) DU145 cells treated with compound **1a**; (C) DU145 cells treated with compound **1b**; (D) DU145RST cells treated with compound **1**; (E) DU145RST cells treated with compound **1a**; (F) DU145RST cells treated with compound **1b**; (G) PC3 cells treated with compound **1**; (H) PC3 cells treated with compound **1a**; (I) PC3 cells treated with compound **1b**; (J) PC3RST cells treated with compound **1**; (K) PC3RST cells treated with compound **1a**; (L) PC3RST cells treated with compound **1b**; (M) HUVEC cells treated with compound **1**; (N) HUVEC cells treated with compound **1a**; and (O) HUVEC cells treated with compound **1b**. Cell viability is expressed relative to the corresponding DMSO-treated control (0 μM). IC_50_ values were calculated by nonlinear regression of the dose–response data and are presented in their respective panels. Data are presented as mean ± SD from three independent experiments (*n* = 3). Statistical significance compared with the corresponding DMSO-treated control: *p < 0.05, **p < 0.01, ***p < 0.001, and ****p < 0.0001.

### 3.3 Antiproliferative and Cell Cycle-Modulating Effects of Compounds **1**, **1a**, and **1b**

To investigate the functional consequences of compounds **1**, **1a**, and **1b** on prostate cancer cells, we evaluated their effects on cell proliferation and cell cycle distribution in parental and DTX-resistant models. The compounds significantly reduced the number of DU145, DU145RST, PC3, and PC3RST cells in a time-dependent manner (Figure 5).

**Figure 5:**
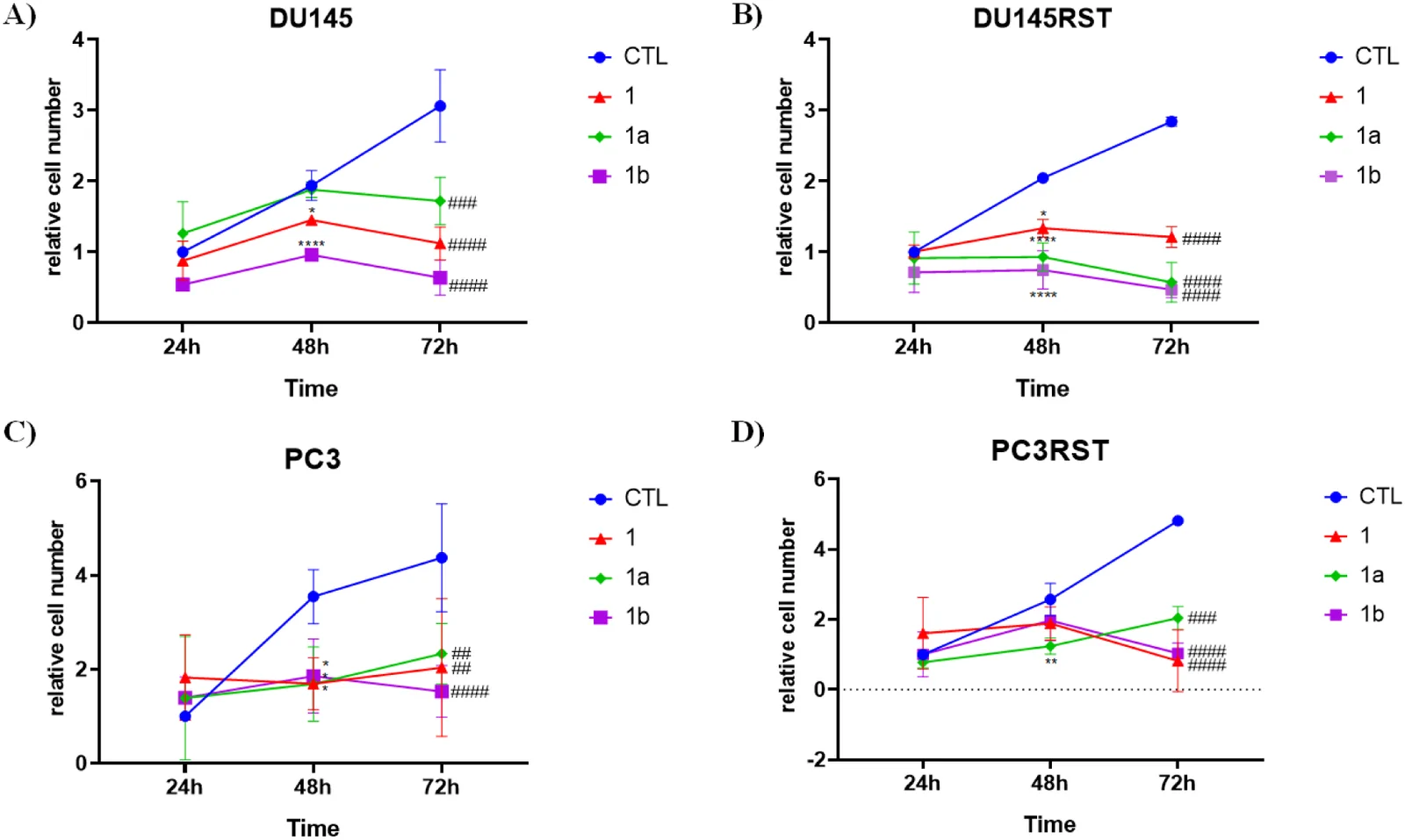
Cell proliferation curves of prostate cancer cells following treatment with compounds **1, 1a**, and **1b**. Parental and docetaxel-resistant prostate cancer cells were treated with the corresponding IC_50_ concentration of each compound, and viable cells were counted after 24, 48, and 72 h using the trypan blue exclusion assay. The panels represent (A) DU145, (B) DU145RST, (C) PC3, and (D) PC3RST cells. Cell numbers are expressed as fold change relative to the control at 24 h, which was set to 1.0. CTL indicates the corresponding untreated or vehicle-treated control. Data are presented as mean ± SD from three independent experiments (*n* = 3). Asterisks indicate statistical significance compared with the corresponding control at 48 h: *p < 0.05, **p < 0.01, ***p < 0.001, and ****p < 0.0001. Number signs indicate statistical significance compared with the corresponding control at 72 h: #*p* < 0.05, ##*p* < 0.01, ###*p* < 0.001, and ####*p* < 0.0001.

To better understand the mechanisms of growth inhibition, we performed cell cycle analyses by flow cytometry after treating the cells for 48 h at the IC_50_ concentration of each compound. Compounds **1**, **1a**, and **1b** altered the cell cycle distribution in both prostate cancer cell lines, with a slightly more pronounced effect in the resistant cell lines. In DU145 cells, treatment with compound **1** reduced the G0/G1 phase population from 75% to 64% and increased the S phase from 5% to 10% and the G2/M phase from 20% to 26% (Figure 6A–E). Compound **1a** reduced the G0/G1 phase population further, to 54%, while increasing the G2/M phase population to 38%. Compound **1b** maintained the G0/G1 phase population at 53% and increased the G2/M phase population to a comparable level, 38%. DU145RST cells exhibited a similar trend, with decreased G0/G1 levels and increased S and G2/M levels in response to the compounds (Figure 6F–J).

**Figure 6:**
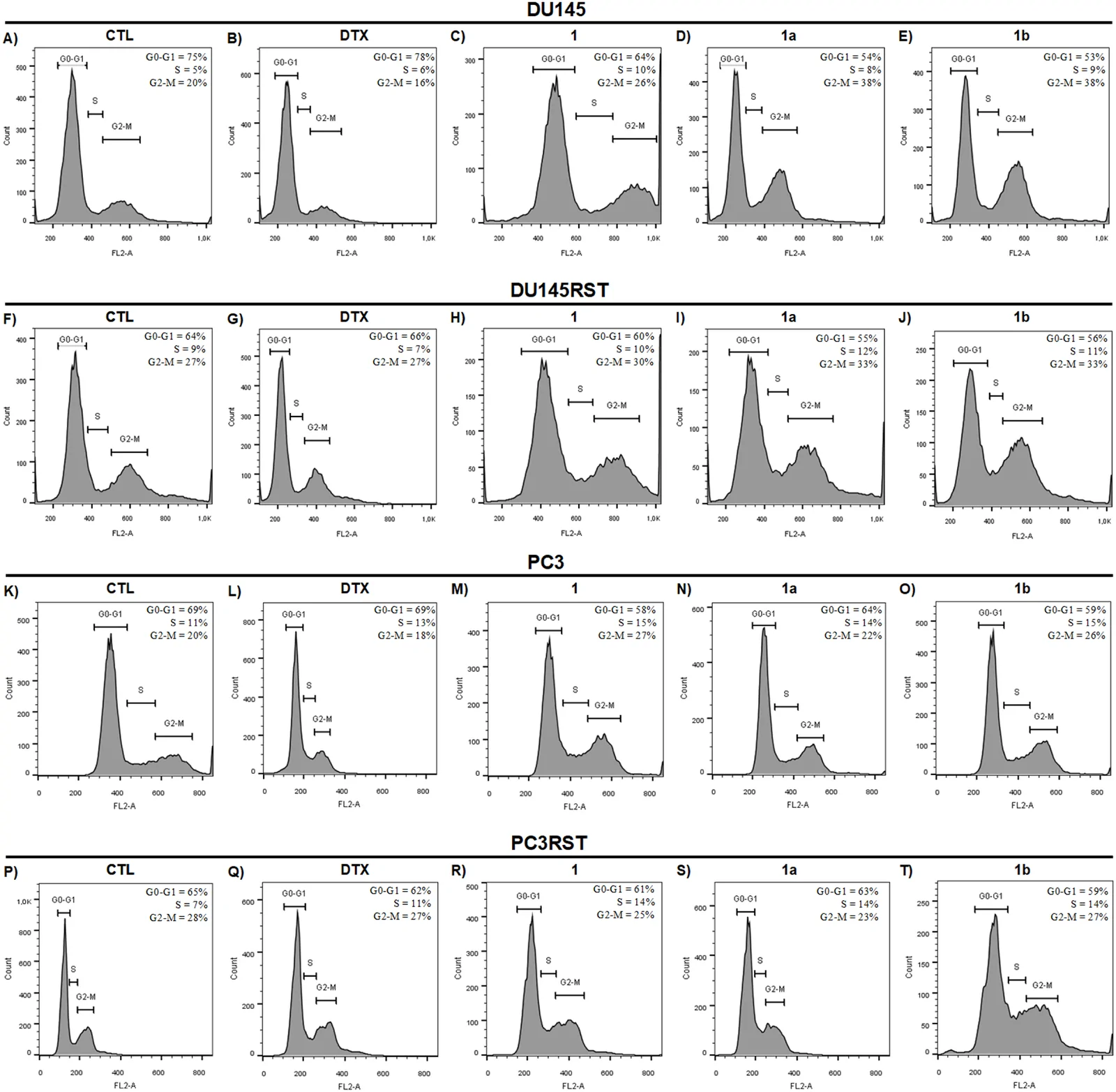
Cell cycle distribution of prostate cancer cells following treatment with compounds **1, 1a**, and **1b.** DU145, DU145RST, PC3, and PC3RST cells were treated for 48 h with docetaxel (DTX) or the respective IC_50_ concentration of compounds **1**, **1a**, and **1b**. Cells were stained with propidium iodide (PI), and DNA content was analyzed by flow cytometry. The panels represent (A–E) DU145 cells: (A) control (CTL), (B) DTX, (C) compound **1**, (D) compound **1a**, and (E) compound **1b**; (F–J) DU145RST cells: (F) CTL, (G) DTX, (H) compound **1**, (I) compound **1a**, and (J) compound **1b**; (K–O) PC3 cells: (K) CTL, (L) DTX, (M) compound **1**, (N) compound **1a**, and (O) compound **1b**; and (P–T) PC3RST cells: (P) CTL, (Q) DTX, (R) compound **1**, (S) compound **1a**, and (T) compound **1b**. Representative DNA content histograms show the percentages of cells in the G_0_/G_1_, S, and G_2_/M phases. The results are representative of three independent experiments.

PC3 cells exhibited similar characteristics (Figure 6K–O): all treatments reduced the G0/G1 population from 69% to between 58% and 64%. The G2/M phase increased with all compounds, reaching 27% with compound **1** and 22%–26% with the derivatives. PC3RST also exhibited reduced G0/G1 levels and increased S and G2/M levels relative to the parental cell line. After treatment with the compounds, the G0/G1 phase remained relatively stable, ranging from 59% to 61%. G2/M levels increased to approximately 25% with all treatments, and modest increases in S phase were observed (Figure 6P–T).

Overall, these results suggest that treatment with compounds **1**, **1a**, and **1b** consistently alters the distribution of cell cycle phases, although the extent of the alteration varies depending on the cell line and resistance status. Increases in the G2/M phase were observed in all models. These shifts suggest that cell cycle arrest is a key antiproliferative mechanism.

### 3.4 Suppression of Clonogenic Potential by Compounds **1**, **1a**, and **1b**

We conducted clonogenic assays to evaluate the long-term effects of treating DTX-sensitive and -resistant prostate cancer cell lines with compounds **1**, **1a**, and **1b**. The cells were treated for 72 h and then left untreated for 15 days to verify their colony-forming capacity. Figure 7 shows that all three compounds significantly inhibited clonogenic capacity in both DU145 and PC3 cell lines, with a stronger effect observed in the resistant cell lines, DU145RST and PC3RST. These results, particularly the comparison of DU145 and DU145RST, reinforce the stronger effect of the compounds on the resistant cell lines and demonstrate their strong cytotoxic properties.

**Figure 7:**
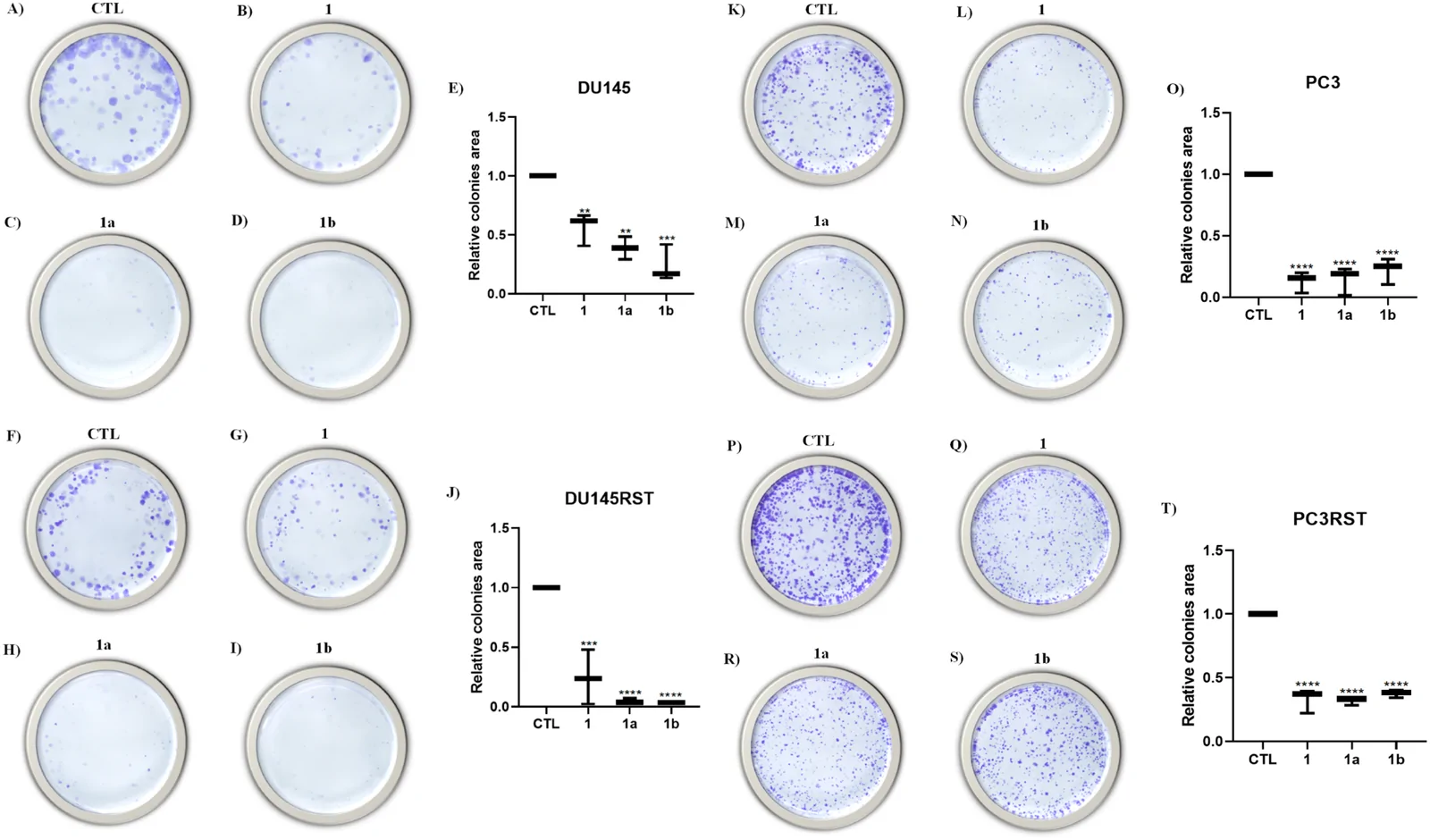
Clonogenic potential of prostate cancer cells following treatment with compounds **1, 1a**, and **1b.** DU145, DU145RST, PC3, and PC3RST cells were treated for 72 h with the respective IC_50_ concentration of each compound and subsequently cultured in compound-free medium for 15 days. Colonies were fixed, stained with crystal violet, and quantified using ImageJ software. Representative images and their corresponding quantifications are shown for (A–E) DU145 cells: (A) control (CTL), (B) compound **1**, (C) compound **1a**, (D) compound **1b**, and (E) relative colony area; (F–J) DU145RST cells: (F) CTL, (G) compound **1**, (H) compound **1a**, (I) compound **1b,** and (J) relative colony area; (K–O) PC3 cells: (K) CTL, (L) compound 1, (M) compound **1a**, (N) compound **1b**, and (O) relative colony area; and (P–T) PC3RST cells: (P) CTL, (Q) compound **1**, (R) compound **1a**, (S) compound **1b**, and (T) relative colony area. Colony areas were normalized to the corresponding control, which was set to 1.0. Quantitative data are presented as mean ± SD from three independent experiments (*n* = 3). Statistical analysis was performed using two-way ANOVA: *p < 0.05, **p < 0.01, ***p < 0.001, and ****p < 0.0001 compared with the corresponding control.

### 3.5 Evaluation of Cell Migration and Cell Death

The prostate cancer cell lines were evaluated for their potential to modulate the cell migration. The cells were treated with mitomycin-c, and a wound-healing assay was performed in the presence of docetaxel, compounds **1**, **1a**, **1b**, or vehicle. None of the compounds altered the wound closure compared to the control group (Supplementary Figure S2).

The cells were also treated with the compounds in all the prostate cancer cell lines and evaluated in the presence of Annexin V-FITC/7-AAD and analyzed in a cell cytometer. Only compound **1a** induced a slight increase in cell death in the DU145 and DU145 RST cell lines but not in the PC3 or the PC3 RST cell lines (Supplementary Figure S3).

### 3.6 Disruption of 3D Spheroid Integrity and Viability in Resistant and Sensitive Models

To analyze the effects of compounds **1**, **1a**, and **1b** in a three-dimensional (3D) model, spheroids were produced from DU145, DU145RST, PC3, and PC3RST cells. The spheroids were treated with the tested compounds at concentrations of 2 × IC_50_ for 72 h. Cell viability was assessed using the PrestoBlue assay, and morphological changes were analyzed by microscopy. As shown in Figure 8, all compounds reduced the viability of the spheroids. Docetaxel-resistant spheroids exhibited a less compact structure and were more sensitive to treatment compared with their parental cell lines. Among the tested compounds, **1** and **1a** significantly reduced the viability of the PC3 cell line, with a more pronounced effect on the resistant spheroids of both cell lines. DU145RST spheroids treated with compound **1b** exhibited viability of less than 40%, compared to approximately 65% for parental DU145 spheroids under the same conditions.

**Figure 8:**
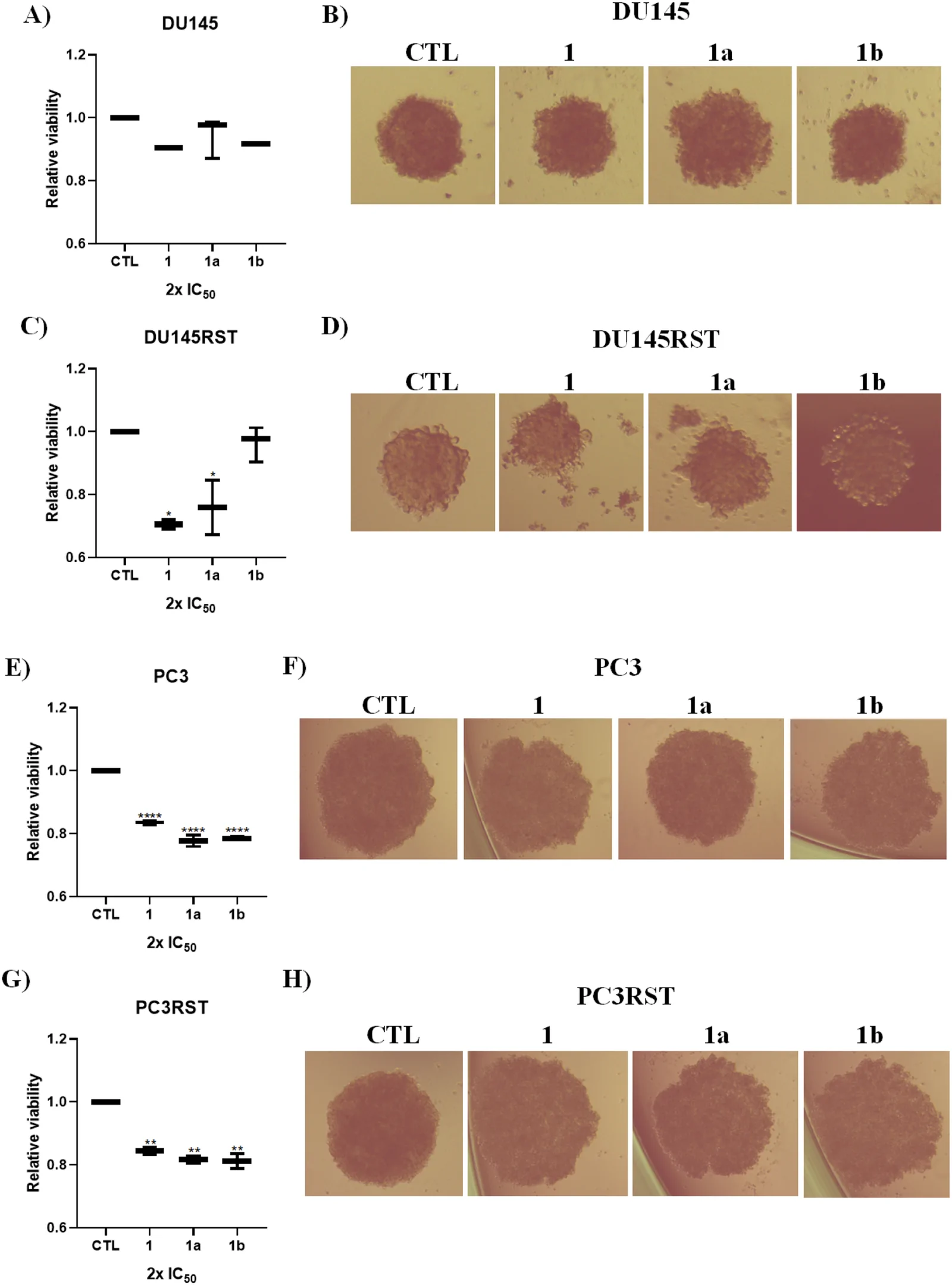
Structural and viability assessment of 3D prostate cancer spheroids following treatment with compounds **1, 1a**, and **1b.** Spheroids formed from DU145, DU145RST, PC3, and PC3RST cell lines were treated for 72 h with compounds **1**, **1a**, and **1b** at 2 × IC_50_ concentrations. (A, C, E, G) Relative viability was assessed using the PrestoBlue assay and is expressed as a relative value of control (DMSO). Quantitative data are presented as mean ± SD of three biological replicates. (B, D, F, H) Representative brightfield images show morphological changes after treatment. Scale bars = 200 μm.

In addition to the decreased viability, the resistant spheroids exhibited notable morphological changes after the treatment. Morphological analysis revealed extensive disaggregation of DU145RST spheroids, particularly in response to compounds **1** and **1a**. The treated spheroids became fragmented and lost cohesion, exhibiting irregular edges and peripheral cell dispersion. These characteristics are indicative of impaired cell-to-cell interactions. Visible morphological changes were observed in both the DU145RST and PC3RST spheroids. When compared with the smooth and compact morphology of untreated PC3 spheroids, PC3RST spheroids showed cell disaggregation and irregular contours after treatment. They also exhibited reduced viability, which reinforced the cytotoxic activity observed in the 2D model.

It is important to note that the effects on the parental cell lines were less pronounced. This once again demonstrates the enhanced activity of these compounds in DTX-resistant cell lines. Although the parental cell lines exhibited reduced cytotoxicity, their morphologies remained intact. Overall, these results reinforce the ability of the tested compounds to penetrate and disrupt three-dimensional tumorlike structures. The selective disaggregation of resistant spheroids indicates a vulnerability unique to these models that could be exploited for therapeutic purposes to overcome chemoresistance.

### 3.7 *In Silico* Prediction of Potential Targets and Binding Affinities for Compounds **1**, **1a**, and **1b**

A reverse pharmacophore mapping analysis was performed to generate a list of protein targets for compounds **1**, **1a**, and **1b**. The top 100 targets for each molecule were ranked by fit score, normalized fit score, and *Z*′-score. Some of these targets were shared by at least two compounds with *Z*′-scores of at least 2.0, which is the limit considered statistically significant in this analysis.^35^ Several of these targets contain proteins that play direct or indirect roles in cell cycle regulation. These targets are summarized in Table 2, which shows the protein name, PDB ID, and *Z*′-scores for each compound.

**Table 2:** Common Predicted Protein Targets for Compounds **1**, **1a**, and **1b** with *Z*′-Score ≥2.0 in At Least One Compound

Target name	PDB ID	1 (*Z*′)	1a (*Z*′)	1b (*Z*′)
Cell division protein kinase 2 (CDK2)	2BTS	3.10	3.86	2.09
Cyclin-A2	1OIU	2.21	2.60	—
Mitogen-activated protein kinase 14 (MAPK14)	2RG6	2.87	2.63	2.38
Carbonic anhydrase II	1G1D	2.39	2.04	3.53
Corticosteroid 11-β-dehydrogenase isozyme 1	3EY4	2.17	2.58	2.13
Prothrombin	1DWD	2.11	—	2.89
Neutrophil collagenase	3DNG	2.06	2.11	—

Among the predicted targets with the highest concordance, cell division protein kinase 2 (CDK2) had the highest *Z*′-score (3.86 for **1a**, 3.10 for **1**, and 2.09 for **1b**). Cyclin A2, a well-known binding partner and regulatory subunit of CDK2 fundamental to the G1/S transition of the cell cycle,^44^ was predicted with a *Z*′-score greater than 2.0 for compounds **1** and **1a**.

Mitogen-activated protein kinase 14 (MAPK14), which is involved in cell cycle checkpoint signaling and cellular stress responses,^45^ was shown to be a common target for all three compounds. *Z*′-scores for this target fluctuated between 2.38 and 2.87. The distribution of *Z*′-scores among these common targets reveals significant overlap between compounds **1** and **1a**, indicating consistent pharmacophoric compatibility among many shared targets. Although structurally different, compound **1b** shares some of these expected targets with slightly lower scores. All three compounds demonstrated high *Z*′-scores with CDK2 and MAPK14, which reinforces their importance in predictive screening and their role in the cell cycle. Complete data, including a list of anticipated targets by compound, fit scores, normalized fit scores, and additional molecular descriptors, are available in Supplementary Table S1.

### 3.8 Molecular Insights into the Action Mechanisms of Compounds **1**, **1a**, and **1b**

The compounds induced cell cycle arrest, and predictive interaction studies supported their role in the cell cycle. Therefore, we evaluated the expression levels of cyclin A2 and CDK2. Figure 9 illustrates the expression levels of cyclin A2 and CDK2 in the DU145 parental and DU145RST DTX-resistant prostate cancer cell lines after treatment with DTX, **1**, **1a**, and **1b**. Compared to the control group, cyclin A2 levels were reduced in all treatment groups, particularly in cells treated with compound **1a**. In the DU145RST subline, CDK2 levels decreased following treatment with DTX and compounds **1** and **1a**. The raw and uncropped western blot images corresponding to Figure 9 are provided in Supplementary Figure S4.

**Figure 9:**
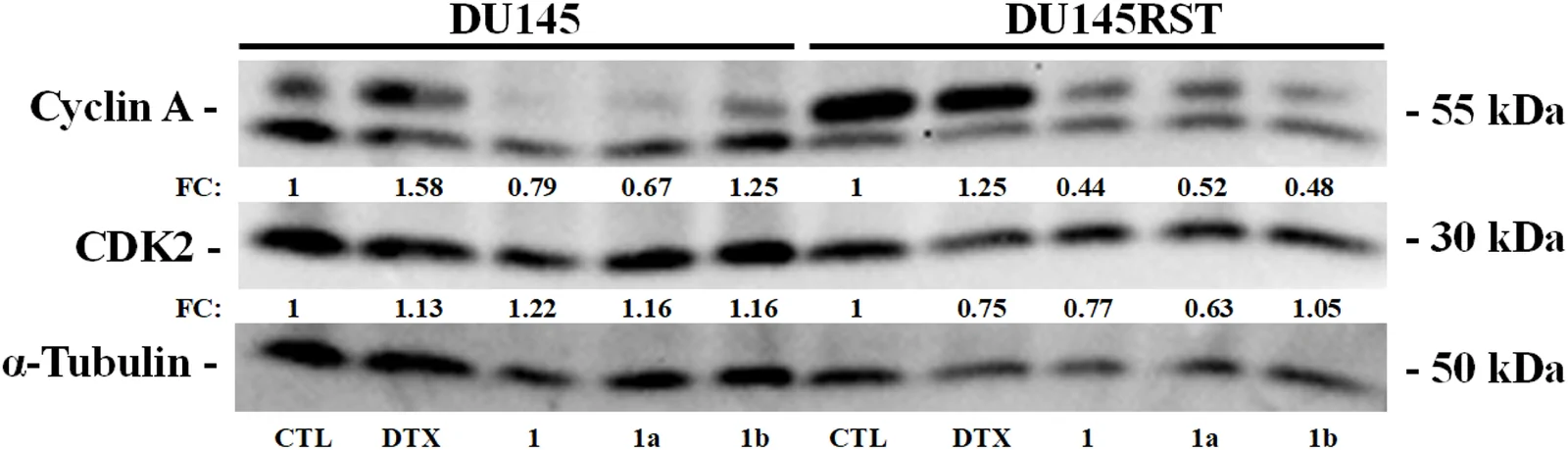
Western blot analysis of cyclin A2 and CDK2 expression. Western blot analysis of cyclin A2 and CDK2 expression in docetaxel-sensitive (DU145) and docetaxel-resistant (DU145RST) prostate cancer cell lines after 48 h of treatment with docetaxel (DTX), **1**, **1a**, and **1b** at their respective IC_50_ concentrations. Tubulin was used as a loading control. Each lane represents a distinct treatment condition, and protein bands correspond to cyclin A2 (∼55 kDa), CDK2 (∼30 kDa), and tubulin (∼50 kDa). FC = fold-change. Representative cropped blots and densitometric quantifications are shown. Raw and uncropped blot images are provided in Supplementary Figure S4.

### 3.9 Molecular Insights of Compounds **1**, **1a**, and **1b** into Inhibition of CDK2 and Cyclin A2

To investigate the molecular mechanisms underlying the antiproliferative effects of compounds **1**, **1a**, and **1b**, molecular docking simulations were carried out, followed by 100 ns molecular dynamics (MD) simulations using the CDK2/cyclin A2 complex (PDB: 1JST), which contains the functional dimer and, therefore, better reflects the *in vivo* complexity of the target.^46^ Two regions of interest relevant to CDK2 regulation were evaluated: the ATP-binding site, located at the catalytic cleft of CDK2,^47^ and the CDK2/Cyclin A2 dimerization interface.^48^ Compounds **1** and **1b** showed a greater energetic tendency for the ATP-binding site, with AutoDock Vina scores of −8.75 and −9.63 kcal/mol, respectively, whereas compound **1a** showed a preferential affinity for the dimerization site, with a score of −8.68 kcal/mol (Figure 10A). This mechanistic distinction guided the simulation strategy: compounds **1** and **1b** were simulated at the catalytic site, while **1a** was simulated at the CDK2/cyclin A2 activation interface. The binding poses predicted by GNINA convolutional neural network (CNN) models (Figure 10B and C), together with the molecular interaction maps at each site (Figure 10D–F), confirm the distinct positioning of each ligand and their steric complementarity with the respective binding environment.

**Figure 10:**
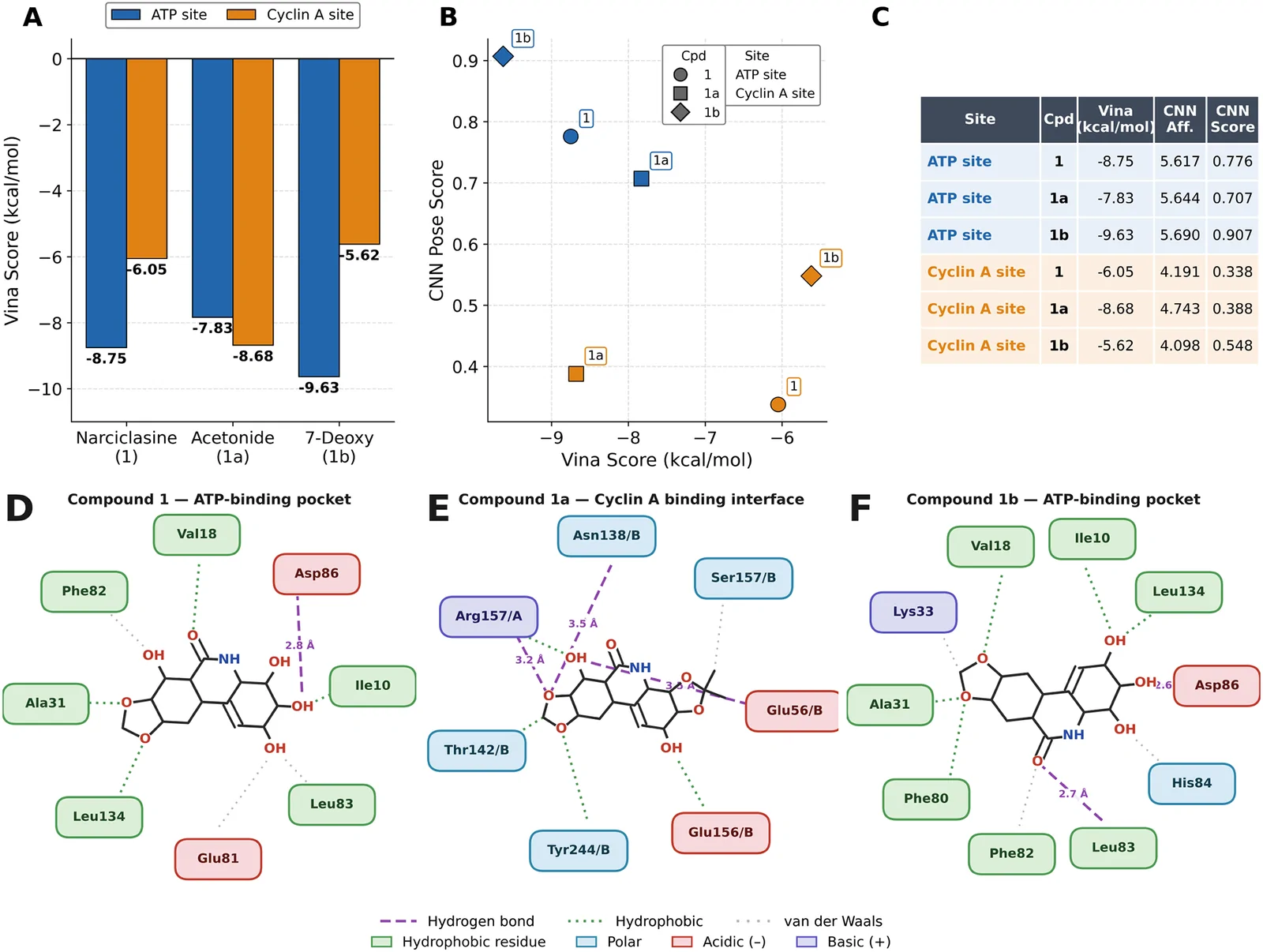
Molecular docking of compounds **1**, **1a**, and **1b** to the CDK2/cyclin A2 complex (PDB: 1JST). (A) Predicted binding free energies at the ATP-binding site (compounds **1** and **1b**) and the CDK2/cyclin A2 dimerization interface (compound **1a**), ranked by AutoDock Vina score (kcal/mol). (B–C) Top-ranked docking poses predicted by the GNINA convolutional neural network (CNN) model, showing the positioning of each ligand within its respective binding environment. (D–F) Two-dimensional molecular interaction maps illustrating the key protein–ligand contacts at the ATP-binding cleft (D, **1**; E, **1b**) and the dimerization interface (F, **1a**), including hydrogen bonds, hydrophobic contacts, and van der Waals interactions.

The analysis of ligand positional RMSD over the 100 ns trajectory confirmed the differential binding stability of each compound within its respective site (Figure 11A). Compound **1** was stabilized with an average RMSD ranging from 0.30 to 0.45 nm, with no detectable unbinding events throughout the simulation. Compound **1b** exhibited the lowest RMSD values during the second half of the trajectory, reaching distances below 0.25 nm after 80 ns, reflecting progressive pose convergence consistent with its highest CNN affinity score among the three compounds (0.907) (Figure 10B and 10 C). In contrast, compound **1a** exhibited greater positional mobility, with RMSD peaks of up to 0.65 nm, a behavior expected for ligands occupying the more open and solvent-exposed dimerization interface compared to the enclosed ATP pocket.^49^ Center-of-mass distance analysis further confirmed the complete retention of compounds **1** and **1b** within the catalytic site (approximately 0.20 nm) throughout the entire simulation (Supplementary Figure S5A).

**Figure 11:**
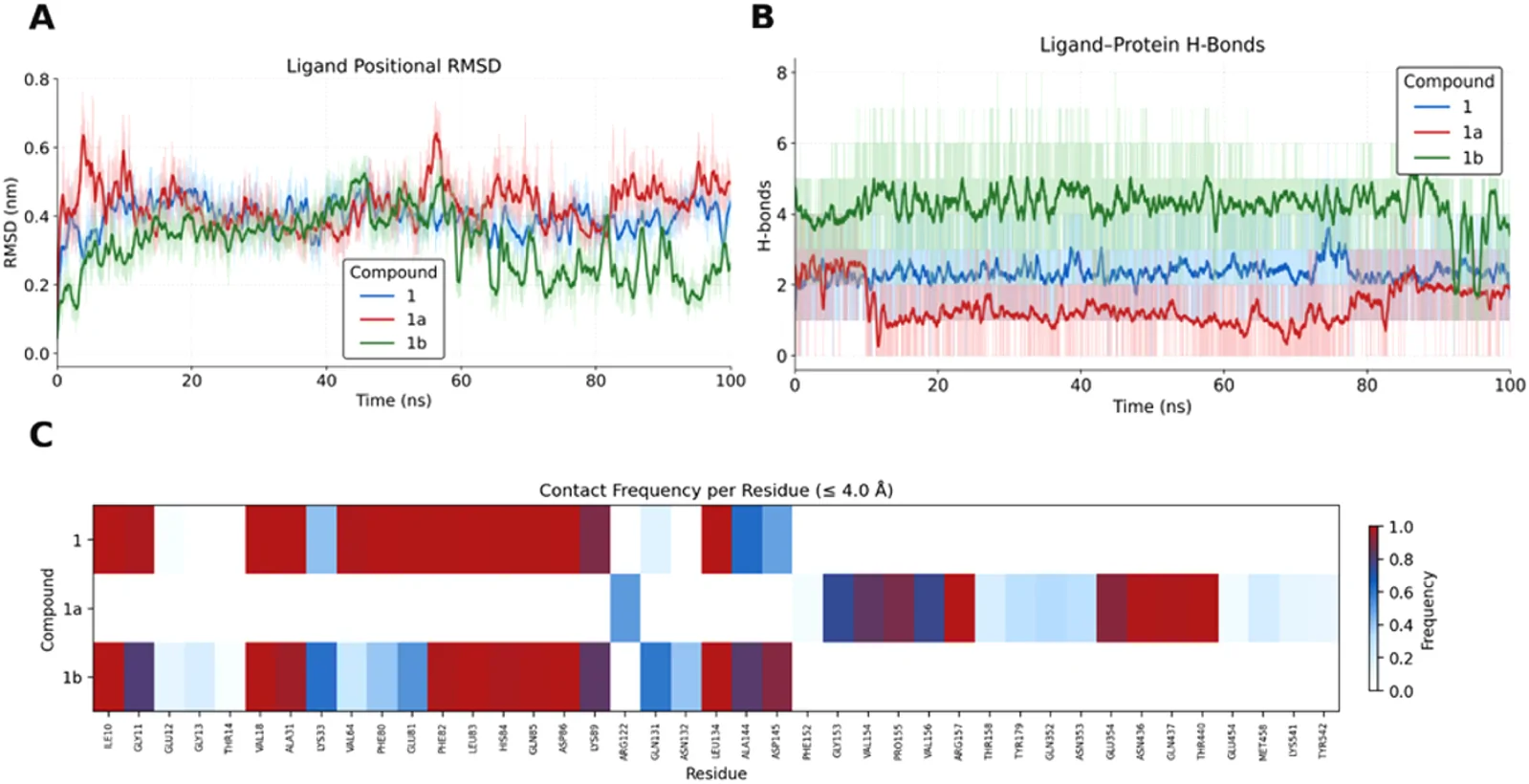
Ligand-binding stability and protein–ligand interaction analysis from 100 ns molecular dynamics simulations. (A) Positional RMSD of the ligand relative to its initial docking pose throughout the simulation, shown for compounds **1** (ATP site), **1a** (dimerization interface), and **1b** (ATP site). (B) Number of direct hydrogen bonds formed between each compound and the CDK2/Cyclin A2 complex over time, calculated using a 3.5 Å donor–acceptor distance cutoff. (C) Residue contact frequency maps showing the proportion of simulation frames in which each protein residue was within 4.5 Å of the bound ligand, for compounds **1**, **1a**, and **1b**.

Hydrogen-bond analysis showed that compound **1b** consistently maintained 4 to 5 hydrogen bonds throughout the 100 ns simulation, outperforming compound **1**, which averaged 2 to 3 hydrogen bonds, and compound **1a**, which formed an average of 1 hydrogen bond (Figure 11B). The higher hydrogen-bonding capacity of compound **1b**, despite the absence of the C-7 hydroxyl group, is explained by the residue contact analysis (Figure 11C): the removal of the bulky C-7 OH reduces steric hindrance at the hinge region, rendering polar residues GLN131, ASN132, GLU12, and THR14 more accessible for direct interaction. Compound **1a**, by contrast, exhibited fewer direct protein hydrogen bonds owing to the acetonide protection of the 3,4-diol, which eliminates two hydrogen-bond donors from the narciclasine scaffold. Accordingly, the energetic stability of **1a** at the dimerization interface is sustained predominantly by hydrophobic and van der Waals contacts with residues ARG157, THR440, ASN436, GLN437, and GLU354, as evidenced by the contact frequency map (Figure 11C). Compounds **1** and **1b** shared the conserved contact core of the CDK2 ATP-binding site comprising LEU134, PHE80, ILE10, VAL18, ALA31, LEU83, ASP86, HIS84, GLN85, PHE82, and GLU81 ^50^ with frequencies approaching 1.0 for the most conserved contacts, validating the predicted binding mode. Compound **1a** displayed an entirely distinct contact profile, engaging residues exclusively at the CDK2/Cyclin A2 interface, thereby confirming the mechanistic distinction predicted by docking. Temporal contact maps for each compound are provided as Supporting Information (Supplementary Figure S5B–D).

MM-GBSA (molecular mechanics/generalized born surface area) was used to calculate the binding free energies, providing a more reliable estimate of protein–ligand binding affinity than docking scores alone because it accounts for conformational flexibility sampled during the MD trajectories and implicit solvation effects. More negative Δ*G*_bind_ values indicate stronger binding. Compound **1b** showed the most favorable Δ*G*_bind_ (−23.3 ± 4.08 kcal/mol), followed by **1a** (−12.9 ± 4.80 kcal/mol) and **1** (−11.3 ± 2.75 kcal/mol) (Figure 12D), indicating that **1b** forms the most stable interaction with CDK2/cyclin A2 among the three compounds. The energy distribution for **1a** showed a pronounced positive tail, reflecting greater conformational flexibility at the interface binding site, while **1** and **1b** showed narrower, more symmetrical distributions, consistent with more stable and geometrically restrained binding poses in the catalytic pocket.

**Figure 12:**
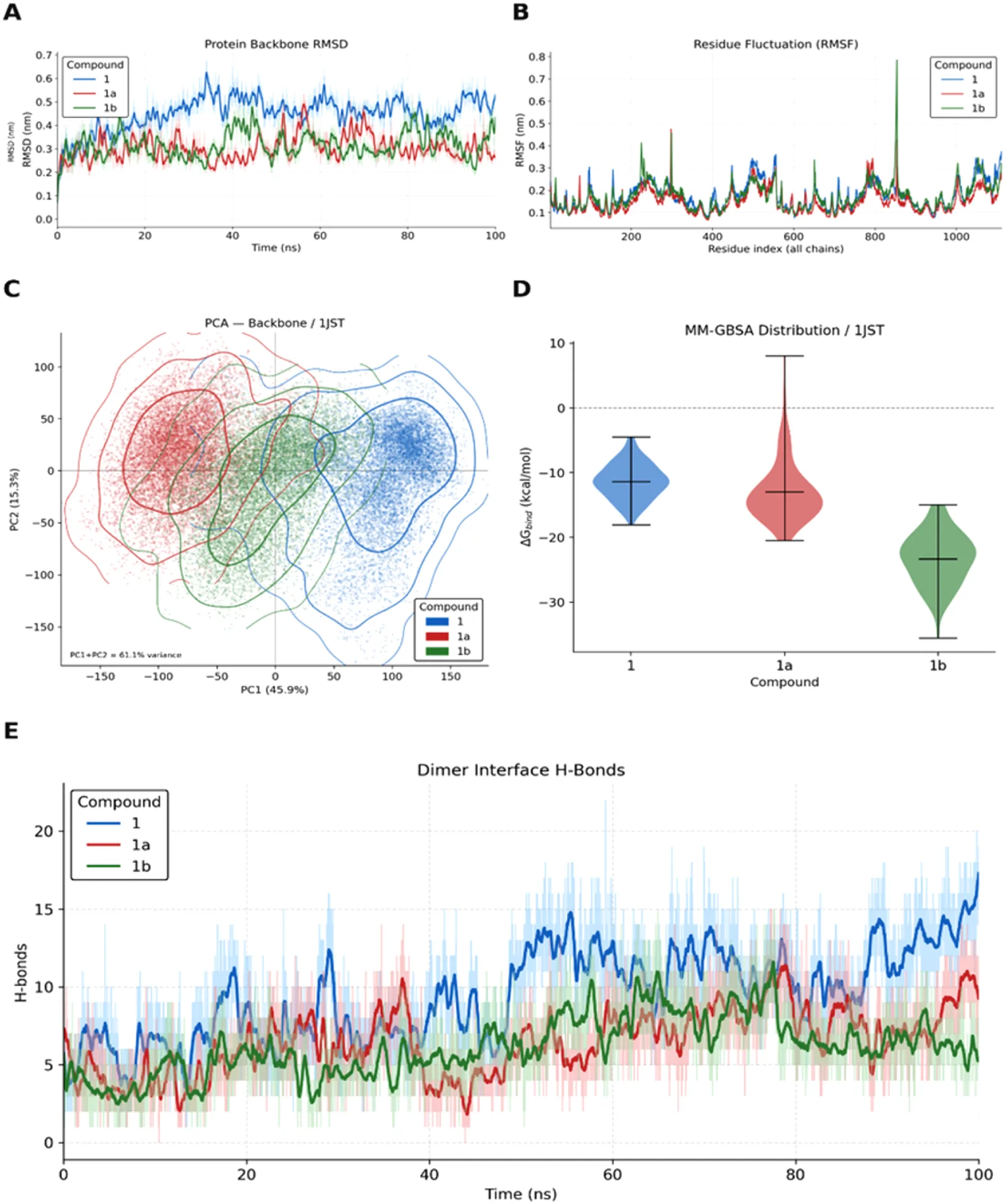
Global conformational dynamics of the CDK2/cyclin A2 complex upon ligand binding. (A) Backbone RMSD of the CDK2/cyclin A2 complex relative to the energy-minimized starting structure over the 100 ns simulation for systems containing compounds **1**, **1a**, and **1b**. (B) Per-residue backbone RMSF, highlighting the differential mobility peak observed exclusively in the C-terminal tail region of CDK2 chain C (residues 296–298) in the presence of compound **1b**. (C) Principal component analysis (PCA) of backbone conformations along PC1, showing the distribution of frames sampled by each system throughout the trajectory. (D) MM-GBSA binding free energy distributions for compounds **1**, **1a**, and **1b** (mean ± SD, kcal/mol). (E) Number of interprotein hydrogen bonds at the CDK2/cyclin A2 dimerization interface over time for each simulated system, illustrating the progressive accumulation of interface contacts induced by compound **1** relative to the stabilized profiles of compounds **1a** and **1b**.

Global complex dynamics were evaluated through backbone RMSD, per-residue RMSF, principal component analysis (PCA), and dynamic cross-correlation maps (DCCM). Compound **1a** induced the lowest overall backbone fluctuation of the three systems, with backbone RMSD stabilizing between 0.25 and 0.35 nm (Figure 12A), consistent with a mechanism of interfacial stabilization that reduces the collective conformational freedom of the dimer. Compound **1** produced the highest conformational mobility, with RMSD reaching up to 0.55 nm after 40 ns (Figure 12A), suggesting allosteric communication from the catalytic pocket to distal regions of the complex through the T-loop and αC helix.^47,50^ Compound **1b** occupied an intermediate position between these two extremes.

Backbone PCA corroborated this segregation: conformations sampled by the system containing compound **1a** were concentrated in the negative region of PC1, whereas the system with compound **1** extensively explored the positive region, reflecting greater conformational heterogeneity (Figure 12C). Compound **1b** showed partial overlap with both distributions, consistent with its dual character as a tight catalytic-site binder that nonetheless propagates allosteric signals across the complex.

Per-residue RMSF analysis revealed a pronounced fluctuation peak exclusive to compound **1b** (reaching approximately 0.79 nm), which was absent in the other systems (Figure 12B). This peak maps exactly onto the C-terminal tail of the second CDK2 molecule in the simulated tetramer (chain C, residues 296–298). This differential mobility indicates that the removal of the C-7 hydroxyl group not only reshapes local interactions within the ATP pocket but also perturbs the extended dynamic communication network between the catalytic site and the distal regions of the complex. This allosteric effect is further supported by the differential DCCM analysis (Supplementary Figure S6), which shows altered correlation patterns between the CDK2 and cyclin A2 residue blocks upon binding of compound **1b**.

Analysis of interprotein hydrogen bonds at the CDK2/cyclin A2 dimerization interface over the course of the simulations revealed a distinctive behavior for compound **1**. While compounds **1a** and **1b** stabilized at approximately 5 to 10 interface hydrogen bonds throughout the trajectory (Figure 12E), compound **1** induced a progressive increase in interface hydrogen bonds, reaching 15 to 17 contacts by the end of the 100 ns simulation (Figure 12E). This observation suggests that occupancy of the ATP-binding site by compound **1** promotes a gradual conformational reorganization of the complex that ultimately consolidates the CDK2/cyclin A2 dimerization interface.

## 4 Discussion

The DU145RST and PC3RST cell lines were generated through stepwise increases in the docetaxel concentration. This yielded models with distinct resistance phenotypes, as demonstrated by cell viability assays and the IC_50_ values. Dose–response curves revealed a significant difference between the parental and resistant cells: parental DU145 and PC3 cells exhibited cytotoxic responses with IC_50_ values of approximately 3 nM, whereas the resistant sublines required approximately twice this concentration to achieve the same effect, meeting or exceeding the commonly used 2-fold threshold of chemoresistance.^51,52^

This phenotype was further supported by molecular alterations affecting the survival and proliferation signaling. Specifically, western blot analyses revealed increased levels of total mTOR and S6K1, accompanied by decreased levels of S6K1 phosphorylation and increased levels of both total and phosphorylated S6. These findings are consistent with well-established mechanisms of docetaxel resistance, including activation of the PI3K/AKT/mTOR axis and enhancement of the protein synthesis machinery.^6,20,53^ Concurrently, increased p62 levels and altered LC3B-II expression suggested dysregulation of autophagic activity, which may contribute to resistance by limiting the drug-induced cell death. Together, these data support the use of DU145RST and PC3RST as robust models for investigating taxane resistance in prostate cancer and evaluating new therapeutic approaches.

Beyond PC3 and DU145, docetaxel-resistant prostate cancer models have been generated from 22Rv1, LNCaP, and C4-2B cells through gradual dose escalation, as well as from xenografts subjected to in vivo chemotherapeutic pressure.^6,14,74–76^ Across these models, resistance-associated features include increased ABCB1/P-glycoprotein-mediated drug efflux, altered β-tubulin isoform expression, androgen receptor splice variants, epithelial-to-mesenchymal transition, enhanced PI3K/AKT/mTOR signaling, and metabolic adaptation.^6,8,14,20,53–55^ Independent laboratories have also established docetaxel-resistant PC3 and DU145 sublines using stepwise dose escalation or prolonged drug exposure, reporting molecular alterations broadly consistent with those observed in our DU145RST and PC3RST sublines.^6,8,14,20,53^ Collectively, these findings position DU145RST and PC3RST within the spectrum of established docetaxel-resistance models while reinforcing the multifactorial nature of resistance and the importance of complementary experimental systems for evaluating novel therapeutic strategies. Studies using resistance models have demonstrated the upregulation of survival pathways. DTX-resistant prostate cancer cell lines have demonstrated activation of the PI3K/AKT/mTOR and MAPK signaling pathways, which enhances protein synthesis, stress resilience, and cellular proliferation.^6,20,53^ In addition, suppression of apoptotic pathways, as indicated by increased Bcl-2 levels and reduced caspase activity, promotes an apoptosis-resistant phenotype.^56^ Epithelial-mesenchymal transition (EMT), which enhances migratory capacity and drug tolerance, is another important contributor to docetaxel resistance.^19^ Although EMT markers and cell migration were not directly assessed in the present study, our clonogenic and 3D spheroid assays demonstrated reduced cohesiveness and impaired long-term survival following treatment with narciclasine and its derivatives. Narciclasine also affects actin cytoskeleton dynamics and impairs migration in several other tumor types, suggesting that these compounds may affect EMT-related processes in prostate cancer.^18,19^ The PI3K/AKT and MAPK pathways have also been implicated in docetaxel resistance.^18,41–43^ The increased levels of mTOR and S6 observed in our resistant cells may be consistent with upstream AKT activation, which could contribute to the survival advantage of these cells.

The contribution of autophagy to resistance warrants further investigation. LC3-II is a canonical marker of autophagosome formation, whereas p62 is an adaptor protein that is normally degraded during autophagic flux.^22,57,58^ Reduced or unchanged LC3-II levels combined with increased p62 levels in our resistant cell lines suggest a dysregulated or incomplete autophagy process in which cargo recognition is increased, but degradation is impaired.^22,57^ This pattern may reflect a shift from cytotoxic autophagy toward a cytoprotective, prosurvival role that protects cells against docetaxel-induced stress. Similar observations have been reported in colorectal and lung cancers, in which inefficient autophagic flux promotes drug tolerance.^22,57^ Consequently, the altered LC3-II/p62 dynamics observed in DU145RST and PC3RST cells further supports the resistant phenotype and highlights autophagy as a possible therapeutic target.

Narciclasine (**1**), a naturally occurring alkaloid from the Amaryllidaceae family, operates via unconventional pathways. Specifically, it inhibits protein synthesis by interacting with eEF1A2,^59,60^ disrupts the actin cytoskeleton,^60^ and modulates stress response pathways.^29,30,61^ These mechanisms may explain its activity against resistant cells, which often evade microtubule-targeting agents through altered tubulin isotype expression, increased drug efflux, and activation of survival pathways.^6,12,14^ Compounds **1**, **1a**, and **1b** exhibited tumor-selective cytotoxicity. Notably, compound **1b** reduced the viability of DU145RST cells while preserving nontumorigenic HUVEC cells, suggesting a favorable selectivity profile. Similarly, compound **1a** exhibited lower potency overall but was more effective against PC3RST cells than against the corresponding parental cells, suggesting that resistance phenotypes may confer exploitable vulnerabilities that can be targeted with these compounds. These vulnerabilities may result from metabolic rewiring, increased oxidative stress, or signaling alterations commonly observed in taxane-resistant prostate cancer.^21,22^ These findings suggest that structural modifications of narciclasine (**1**) may enhance activity against resistant cancer cells without proportionally increasing cytotoxicity toward nontumorigenic cells.

Compounds **1**, **1a**, and **1b** reduced the total number of viable cells, particularly in the resistant cell lines. Compound **1b** exerted the strongest antiproliferative effect on DU145RST cells, causing a significant decrease in the G0/G1 population and an increase in the S and G2/M populations. These results are consistent with previous studies demonstrating the ability of narciclasine to induce cell cycle arrest in the G1 or G2/M phase, depending on the cellular context,^59,62,63^ and which are often associated with alterations in cyclin-dependent kinases (CDKs) and increased levels of inhibitors such as CDKN1A.^63,64^ Furthermore, all compounds exhibited potent clonogenic potential inhibition, reducing both the colony number and size, which further validates their impact on sustained proliferation. These effects were confirmed in the 3D spheroid model, which more accurately reflects the in vivo tumor architecture. The compounds substantially impaired spheroid growth and integrity, exhibiting increased effectiveness in DU145RST cells compared to parental cells. Chemoresistant cells have been described as more susceptible to agents that disrupt the cytoskeleton, protein synthesis, or stress adaptation.^21,65^ The improved response observed in resistant spheroids, despite the limitations of drug penetration and hypoxia in 3D models,^66–69^ strengthens the evidence that these compounds retain or even enhance their antitumor activity under more physiologically relevant conditions.

Pharmacophore-based prediction using the PharmMapper server provided mechanistic hypotheses regarding the activities of the compounds. Reverse pharmacophore mapping identified the critical G1/S transition regulators CDK2 and cyclin A2 were identified as potential targets. These targets were identified with high confidence among the three tested compounds with Z-scores exceeding 20. This prediction aligns well with the experimentally observed G2/M arrest and suggests a mechanism rooted in the inhibition of CDK2–cyclin complexes, which are essential for initiating DNA synthesis.^70–72^ The observed phenotypes, including reduced proliferation, impaired colony formation, and suppressed spheroid growth, are consistent with CDK2 inhibition. These findings reinforce the connection between computational predictions and actual biological processes.^71–73^ Furthermore, western blot analysis demonstrated that treatment with these compounds modulated CDK2 and cyclin A2 protein levels.

Molecular docking and MD simulations provided a mechanistic framework for interpreting the differential modulation of CDK2 and cyclin A2 protein levels observed by western blot. The three compounds occupy distinct binding sites on the CDK2/cyclin A2 complex: compounds **1** and **1b** occupy the ATP-binding catalytic cleft, and compound **1a** occupies the CDK2/cyclin A2 dimerization interface. Each predicted binding mode was associated with specific conformational and thermodynamic effects on the complex, which may help to explain the experimentally observed differences in protein expression.

The greatest reduction in cyclin A2 levels in DU145 cells was induced by compound **1a**. This result is mechanistically anticipated by the compound’s preferential binding to the protein–protein interaction (PPI) interface. Disrupting the dimerization interface destabilizes the CDK2/cyclin A2 heterodimer and exposes the cyclin subunit to proteasomal degradation. This directly reduces its steady-state abundance without requiring catalytic inhibition.^48^ MD data support this scenario at the atomic level. Compound **1a** maintained high-frequency contacts with interface residues ARG157, THR440, ASN436, GLN437, and GLU354 while inducing the lowest overall backbone root-mean-square deviation (RMSD) of the three systems (0.25–0.35 nm). This is consistent with stabilization of the dimer in a disassembly-competent conformation. Acetonide protection of the C-3,4-diol eliminates two hydrogen-bond donors from the narciclasine scaffold. This key structural modification may redirect the binding preference of the compound toward the more open and hydrophobic PPI surface by reducing its capacity to form direct hydrogen bonds with hinge-region residues.^74^

In the DU145RST, the most notable suppression of cyclin A2 was observed following treatment with compound **1**, whereas CDK2 levels decreased following treatment with DTX, compound **1**, or compound **1a** but were restored following treatment with compound **1b**. The recovery of CDK2 protein in the presence of sustained cyclin A2 suppression by compound **1b** is one of the most informative observations in the data set. This finding is fully consistent with the compound’s ATP-competitive binding mode. Among the three compounds, compound **1b** bound the catalytic cleft with the highest affinity and maintained near-unity contact frequencies, sustaining four to five hydrogen bonds throughout the 100 ns simulation. Catalytic-site inhibitors of this class suppress kinase activity while leaving the kinase–cyclin assembly intact because the cyclin binding surface is spatially remote from the ATP pocket and is not directly perturbed.^47^ Therefore, CDK2 protein remains stable at the translational level while being rendered catalytically inert. The fact that this behavior emerges specifically in the resistant subline suggests that DU145RST cells may rely on CDK2 protein stability as part of their adaptive response. Compound **1b** may therefore uncouple this axis by suppressing kinase activity without triggering CDK2 degradation.

The distinct cyclin A2 suppression profile of compound **1** in DU145RST cells, the strongest among all treatments in this context, is further clarified by analysis of the MD trajectories. Compound **1**, uniquely among the three, induced a progressive accumulation of interprotein hydrogen bonds at the CDK2/cyclin A2 dimerization interface, reaching 15 to 17 contacts by the end of the 100 ns simulation, compared to 5 to 10 for compounds **1a** and **1b**. This trajectory suggests that ATP-pocket occupancy by compound **1** propagates allosteric signals that gradually tighten the dimerization interface, which could lock the complex in a conformation that is catalytically inactive yet physically intact in the short term. Over the time scale of a cellular experiment, however, a kinase–cyclin complex that remains locked in an inactive conformation could be targeted for degradation by quality-control pathways that recognize catalytically unproductive assemblies, ultimately reducing steady-state cyclin A2 levels.^50^

Collectively, the MD simulations predict, and the western blot data confirm, that compounds **1**, **1a**, and **1b** suppress the CDK2/cyclin A2 axis through three mechanistically distinct routes: allosteric interface tightening leading to cyclin A2 degradation over extended time scales (compound **1**), direct PPI disruption causing acute cyclin A2 destabilization (compound **1a**), and ATP-competitive catalytic inhibition preserving CDK2 protein stability while eliminating kinase activity (compound **1b**). This nonredundant mechanistic diversity, arising from structural modifications within the same Amaryllidaceae isocarbostyril scaffold, may explain both the consistent antiproliferative activity observed in parental and docetaxel-resistant models and the compound-specific protein expression patterns that emerge in cells in which survival adaptations had reshaped the CDK2/cyclin A2 regulatory landscape.

The present study demonstrated that compounds **1**, **1a**, and **1b** effectively inhibited prostate cancer proliferation and cell cycle progression and generally exhibited greater potency in resistant cells. These results suggest the presence of exploitable vulnerabilities associated with the chemoresistance state. The selectivity of these compounds, the mechanistic support provided by *in silico* analyses, and their broad activity across different experimental models make them promising therapeutic candidates. These compounds may therefore help to overcome docetaxel resistance and expand the therapeutic options available for refractory prostate cancer.

## 5 Conclusion

The docetaxel-prostate cancer models were robust and reliable, mirroring the crucial molecular and functional traits described in the literature. These features are extensively documented in the academic literature, including the activation of survival pathways and significant changes in autophagic flux. The validity of these models provides a solid foundation for thoroughly investigating the processes underlying DTX resistance and testing novel treatments. Using these models, our research revealed that compounds **1**, **1a**, and **1b** exhibit potent antiproliferative effects. These effects were evident against both docetaxel-sensitive and -resistant prostate cancer cells. Viability, cell cycle, clonogenic, and 3D spheroid assays demonstrated that the aforementioned compounds not only curb cell growth but are also more effective at suppressing chemoresistant cells. This enhanced potency, coupled with minimal impact on nontumorigenic cells, underscores their selective cytotoxicity and therapeutic potential. Furthermore, G2/M cell cycle arrest and prolonged suppression of proliferative capacity were validated by a western blot assay and in silico target prediction. These results indicated that CDK2 and cyclin A2 are molecular targets, and that the modifications introduced to the original molecule altered the compounds’ binding and inhibitory activity to these cellular targets. Overall, these results imply that narciclasine-based compounds might effectively overcome taxane resistance.

## Supplementary Material



## Notes

Figure S1: Raw and uncropped Western blot images corresponding to Figure 3; Figure S2: Wound-healing assay to evaluate the effects of the compounds on cell migration; Figure S3: Cell death mediated by the compounds; Figure S4: Raw and uncropped Western blot images corresponding to Figure 9; Figure S5A: Center-of-mass distance between each ligand and its respective binding site along the 100 ns MD simulation, S5B: Temporal contact map—compound **1**(≤4.0 Å), S5C: Temporal contact map—compound**1a**(≤4.0 Å), S5D: Temporal contact map—compound **1b**(≤4.0 Å); Figure S6: Differential dynamic cross-correlation maps (ΔDCCM) relative to compound **1**; Table S1: Complete data, including the list of predicted targets per compound, fit scores, normalized fit scores for compounds **1**,**1a**, and**1b** (PDF)

## Abbreviations

3D: three-dimensional
ABCB1: ATP-binding cassette subfamily B member 1
ADT: androgen-deprivation therapy
AKT: protein kinase B
ANOVA: analysis of variance
AR: androgen receptor
ATP: adenosine triphosphate
Bcl-2: B-cell lymphoma 2
CC: column chromatography
CCNA2: cyclin A2 gene
CD_3_OD: deuterated methanol
CDK: cyclin-dependent kinase
CDK2: cyclin-dependent kinase 2
ChelpG: charges from electrostatic potentials using a grid-based method
CHARMM: Chemistry at Harvard Macromolecular Mechanics
CNN: convolutional neural network
CRPC: castration-resistant prostate cancer
CTL: control
DCCM: dynamic cross-correlation matrix
DMEM: Dulbecco’s Modified Eagle Medium
DMF: N,N-dimethylformamide
DMSO: dimethyl sulfoxide
DMSO-*d*_6_: deuterated dimethyl sulfoxide
DNA: DNA
DTX: docetaxel
eEF1A2: eukaryotic translation elongation factor 1 alpha 2
EMT: epithelial-to-mesenchymal transition
FBS: fetal bovine serum
FC: fold change
GAPDH: glyceraldehyde-3-phosphate dehydrogenase
GROMACS: GROningen MAchine for Chemical Simulations
HF: Hartree–Fock
HPLC: high-performance liquid chromatography
HRP: horseradish peroxidase
HUVEC: human umbilical vein endothelial cell
IC_50_: half-maximal inhibitory concentration
IgG: immunoglobulin G
LC3B: microtubule-associated protein 1 light chain 3 beta
LINCS: linear constraint solver
MAPK14: mitogen-activated protein kinase 14
MD: molecular dynamics
MM-GBSA: molecular mechanics/generalized Born surface area
mTOR: mechanistic target of rapamycin
MTT: 3-(4,5-dimethylthiazol-2-yl)-2,5-diphenyltetrazolium bromide
NIH: National Institutes of Health
NMR: nuclear magnetic resonance
NPT: constant number of particles, pressure, and temperature
NVT: constant number of particles, volume, and temperature
PBS: phosphate-buffered saline
PC1: principal component 1
PCA: principal component analysis
PCa: prostate cancer
PDB: Protein Data Bank
PI: propidium iodide
PI3K: phosphoinositide 3-kinase
P-gp: P-glycoprotein
PPI: proteinprotein interaction
RMSD: root-mean-square deviation
RMSF: root-mean-square fluctuation
RST: docetaxel-resistant subline designation
S6: ribosomal protein S6
S6K1: ribosomal protein S6 kinase 1
SD: standard deviation
SDS: sodium dodecyl sulfate
SMILES: simplified molecular-input line-entry system
SQSTM1/p62: sequestosome 1/p62
TIP3P: transferable intermolecular potential with three points
TLC: thin-layer chromatography
