# Supplementary material for: Overcoming Docetaxel Resistance in Prostate Cancer by Targeting Cell Cycle Progression with Narciclasine-Based Compounds
Source: ACS Omega. 2026 Sep 2;11(36):54245–65. doi: 10.1021/acsomega.6c05272 (PMC13584155; doi:10.1021/acsomega.6c05272)
Supplement: Supplementary file 1 [file ao6c05272_si_001.pdf]

OVERCOMING DOCETAXEL RESISTANCE IN PROSTATE CANCER BY TARGETING CELL CYCLE PROGRESSION WITH NARCICLASINE-BASED COMPOUNDS

Ranyelison Silva Machado[1,2], Kaio S. Gomes[3], Augusto B. Farias[3],
Natália Meneses Araújo[1,2], Giovanna Barbosa Chanes[1,2], Laura Maria Pinto Dias[1,4], Bianca Zaia Franco Ferreira[4], Carla Cristina Lopes[4], Ileana G. S. Rubio[1,5], Fernando Moreira Simabuco[4], Daniela G. G. Rando[2], Hugo Pequeno Monteiro[6],
Adolfo Garcia Erustes[7], Soraya Soubhi Smaili[7], David Ryffel[8], David Sarlah[8],
Giselle Cerchiaro[3], João Henrique G. Lago[3], Rodrigo Esaki Tamura*[1,2,9]

[1]Cancer Molecular Biology Laboratory, Federal University of São Paulo, São Paulo - SP, Brazil

[2]Posgraduate Program of Chemical Biology, Institute of Environmental, Chemical and Pharmaceutical Sciences, Federal University of São Paulo, Diadema - SP, Brazil

[3]Center for Natural and Human Sciences, Federal University of ABC, 09210-180 Santo Andre - SP, Brazil.

[4]Department of Biochemistry, Federal University of São Paulo, São Paulo – SP, Brazil

[5]Postgraduate Program of Pathology, Postgraduate Program of Biotechnology, Department of Biological Sciences, Federal University of São Paulo, Diadema – SP, Brazil

[6]Department of Biochemistry, Center for Cellular and Molecular Therapy - CTCMol, Federal University of São Paulo, São Paulo - SP, Brazil.

[7]Pharmacology Department, Federal University of São Paulo, São Paulo – SP, Brazil.

[8]Department of Chemistry, Wiess School of Natural Sciences, Rice University, 770055 Houston, TX, USA

[9]Department of Biochemical-Pharmaceutical Technology, Faculty of Pharmacy, University of São Paulo, São Paulo, SP, Brazil

*corresponding author

Supplementary Figure S1. Raw and uncropped Western blot images corresponding to Figure 3.

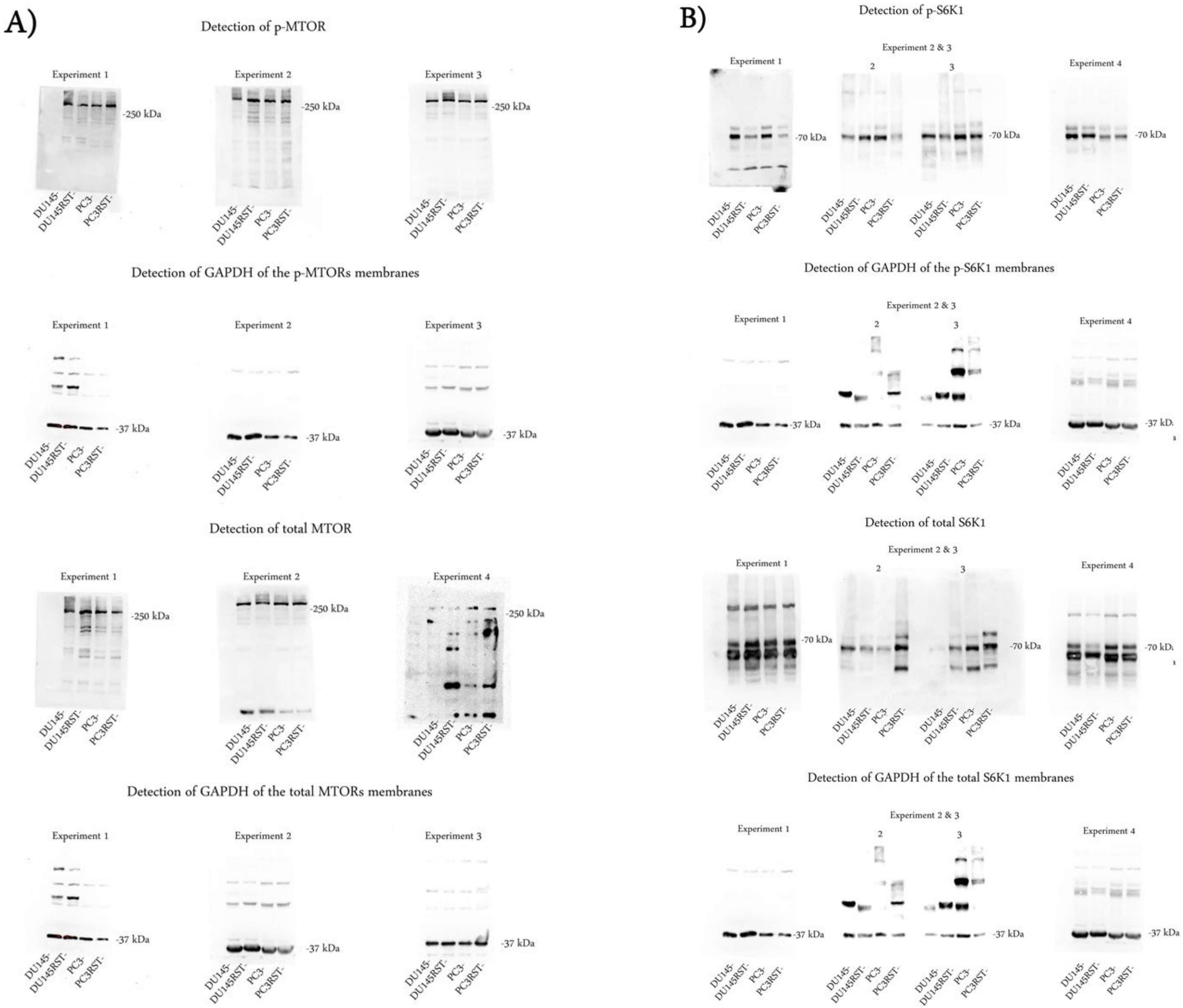

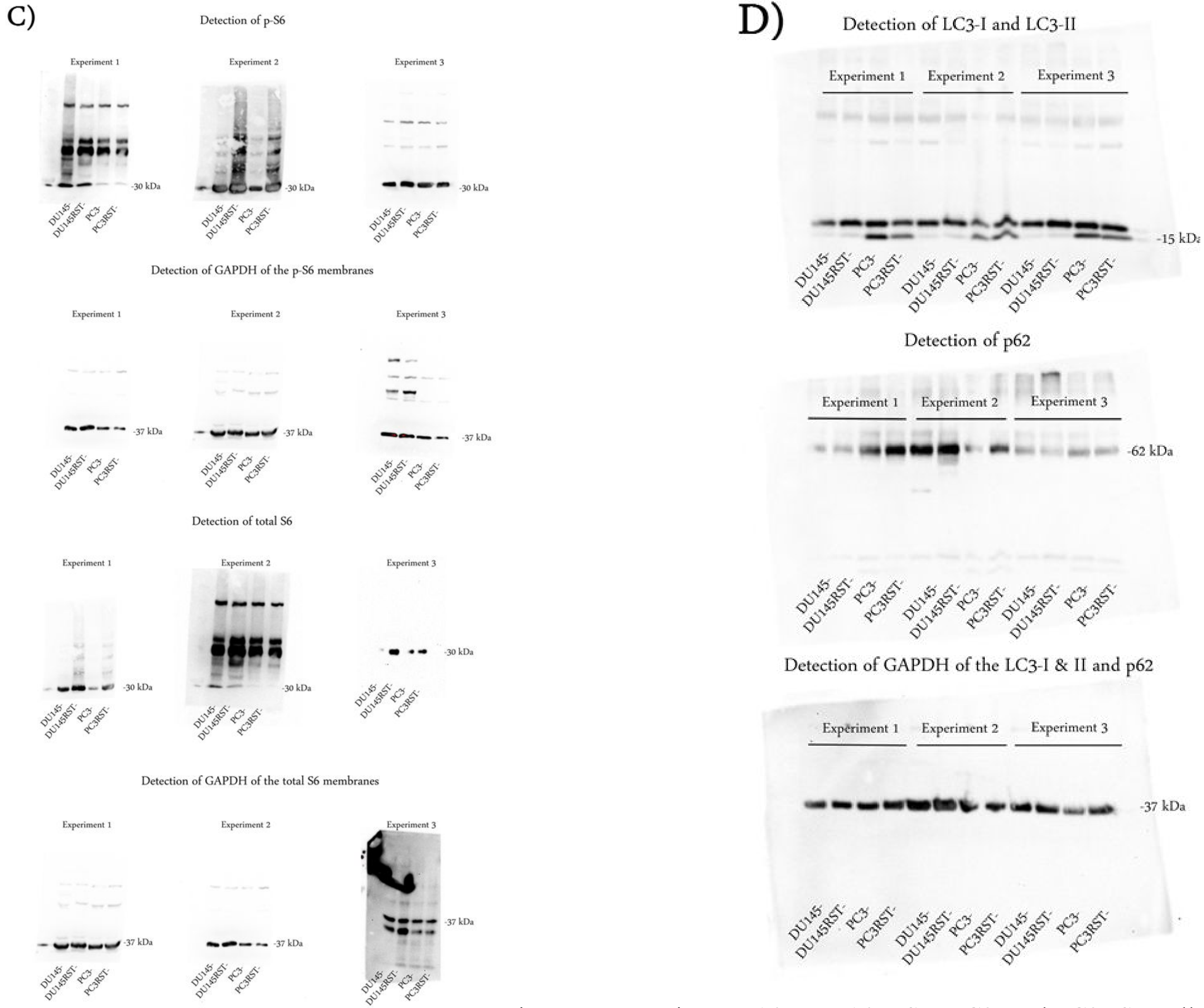

Raw and uncropped membrane images from Western blot experiments comparing DU145, DU145RST, PC3, and PC3RST cells are shown. (A) Phospho-mTOR (p-mTOR) and total mTOR, with the corresponding GAPDH loading controls; (B) phospho-S6K1 (p-S6K1) and total S6K1, with the corresponding GAPDH loading controls; (C) phospho-S6 (p-S6) and total S6, with the corresponding GAPDH loading controls; and (D) LC3B-I/II and p62, with the corresponding GAPDH loading controls. Individual experiments, lane identities, and molecular-mass positions are indicated in each image. The cropped representative blots and densitometric quantifications are presented in Figure 3.

Supplementary Figure S2. Wound-healing assay to evaluate the effects of the compounds on cell migration.

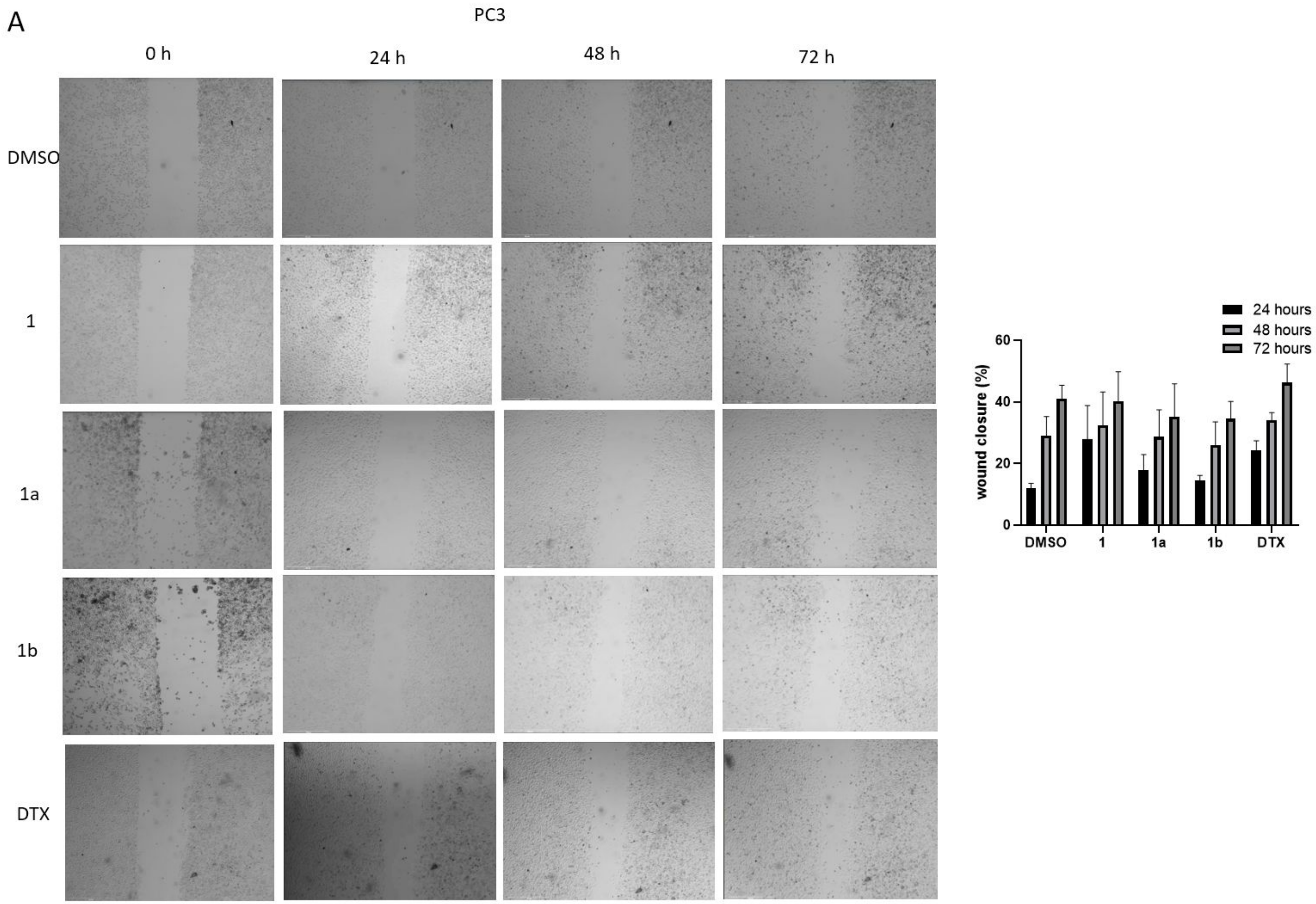

Prostate cancer cell lines, PC3 (A), PC3 RST (B), DU145 (C), DU145 RST (D) were treated with the IC50 of compounds, docetaxel (DTX) or vehicle (DMSO) for up to 72 hours and the wound closure was evaluated. Asterisks indicate statistical significance compared with the corresponding control: **$p < 0.01$.

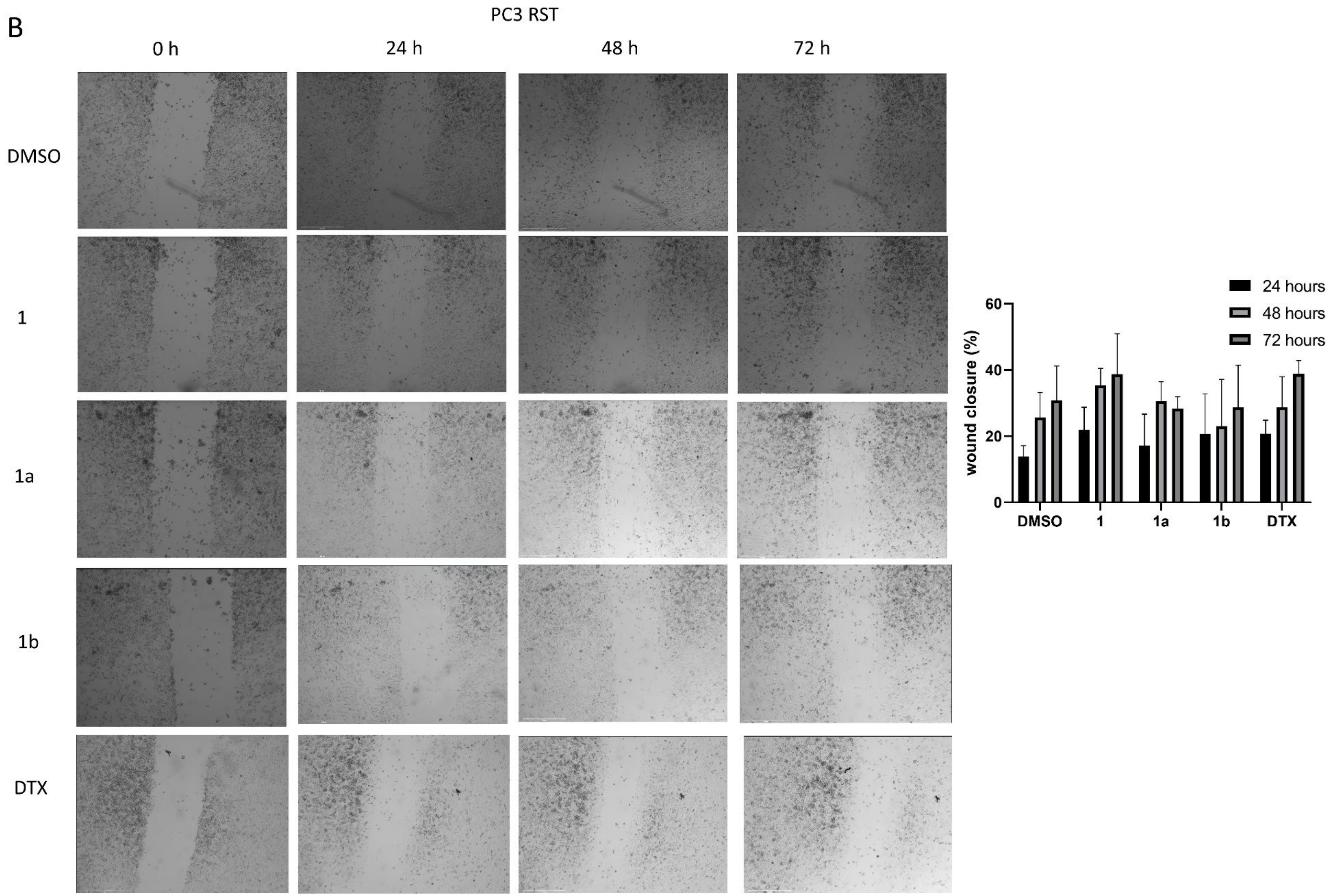
B
PC3 RST
0 h
24 h
48 h
72 h
DMSO
1
1a
1b
DTX
24 hours
48 hours
72 hours
wound closure (%)
0
20
40
60
DMSO
1
1a
1b
DTX

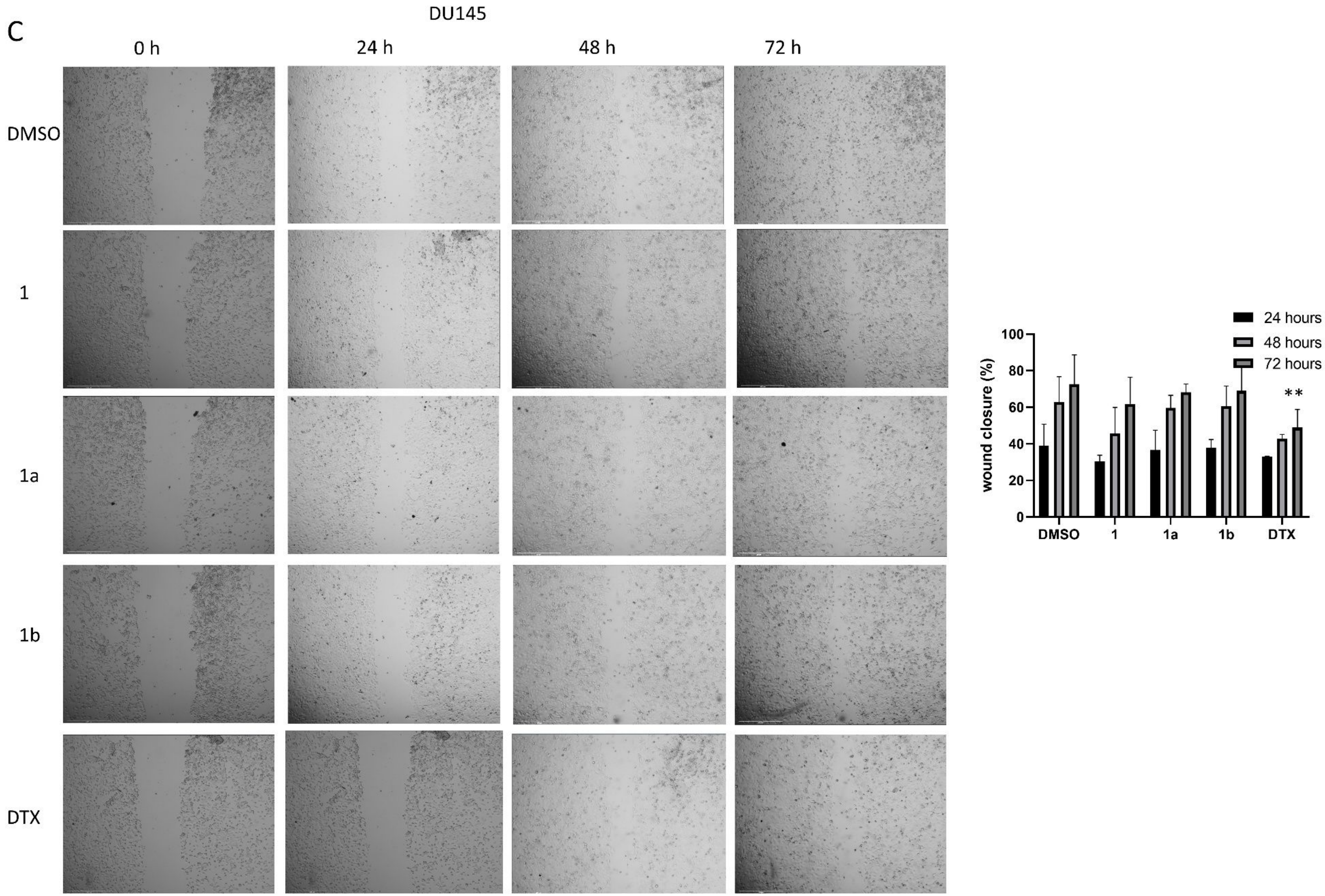
C
DU145
0 h
24 h
48 h
72 h
DMSO
1
1a
1b
DTX
24 hours
48 hours
72 hours
wound closure (%)
100
80
60
40
20
0
**
DMSO
1
1a
1b
DTX

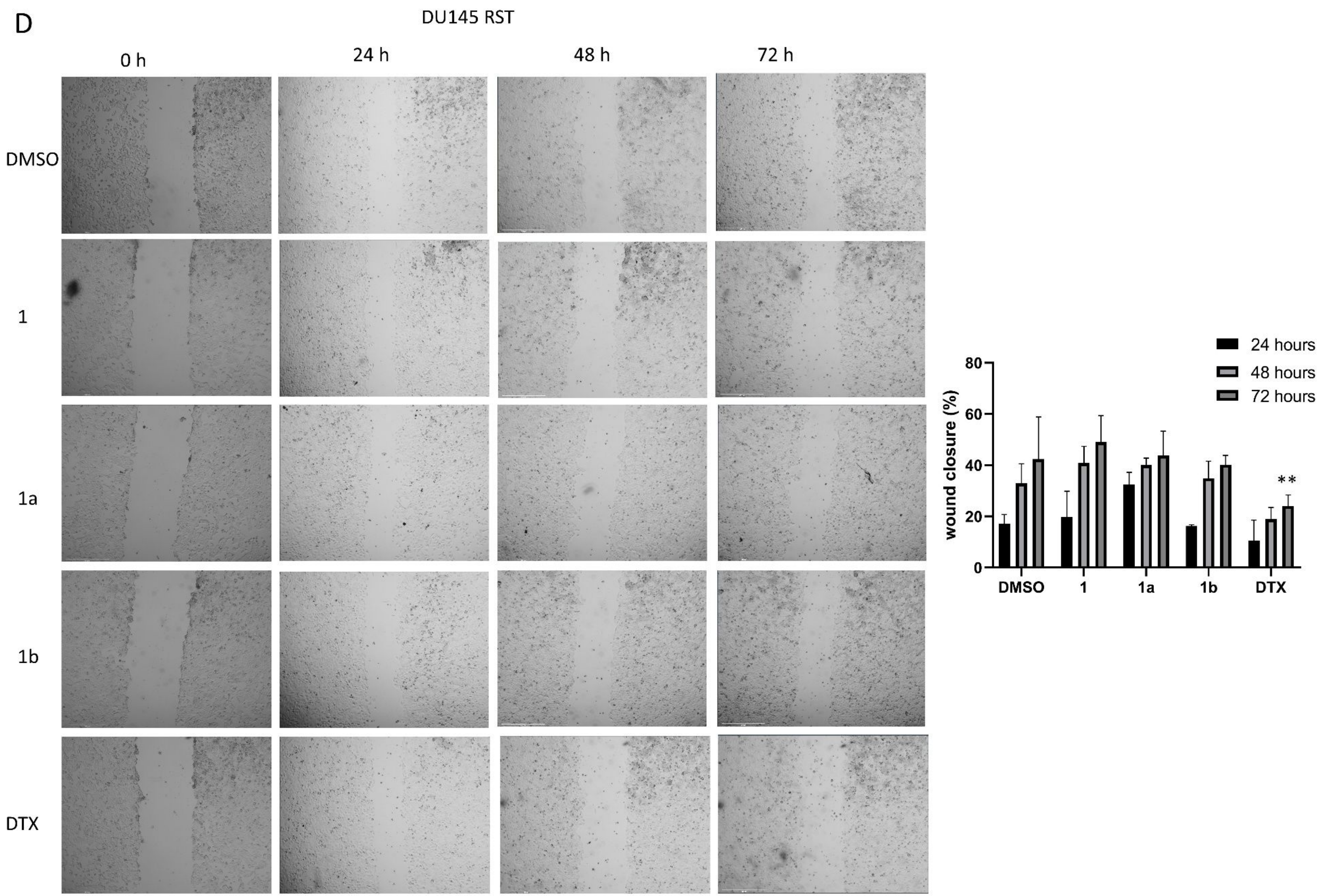
D
DU145 RST
0 h
24 h
48 h
72 h
DMSO
1
1a
1b
DTX
24 hours
48 hours
72 hours
wound closure (%)
80
60
40
20
0
DMSO
1
1a
1b
DTX
**

Supplementary Figure S3. Cell death mediated by the compounds.

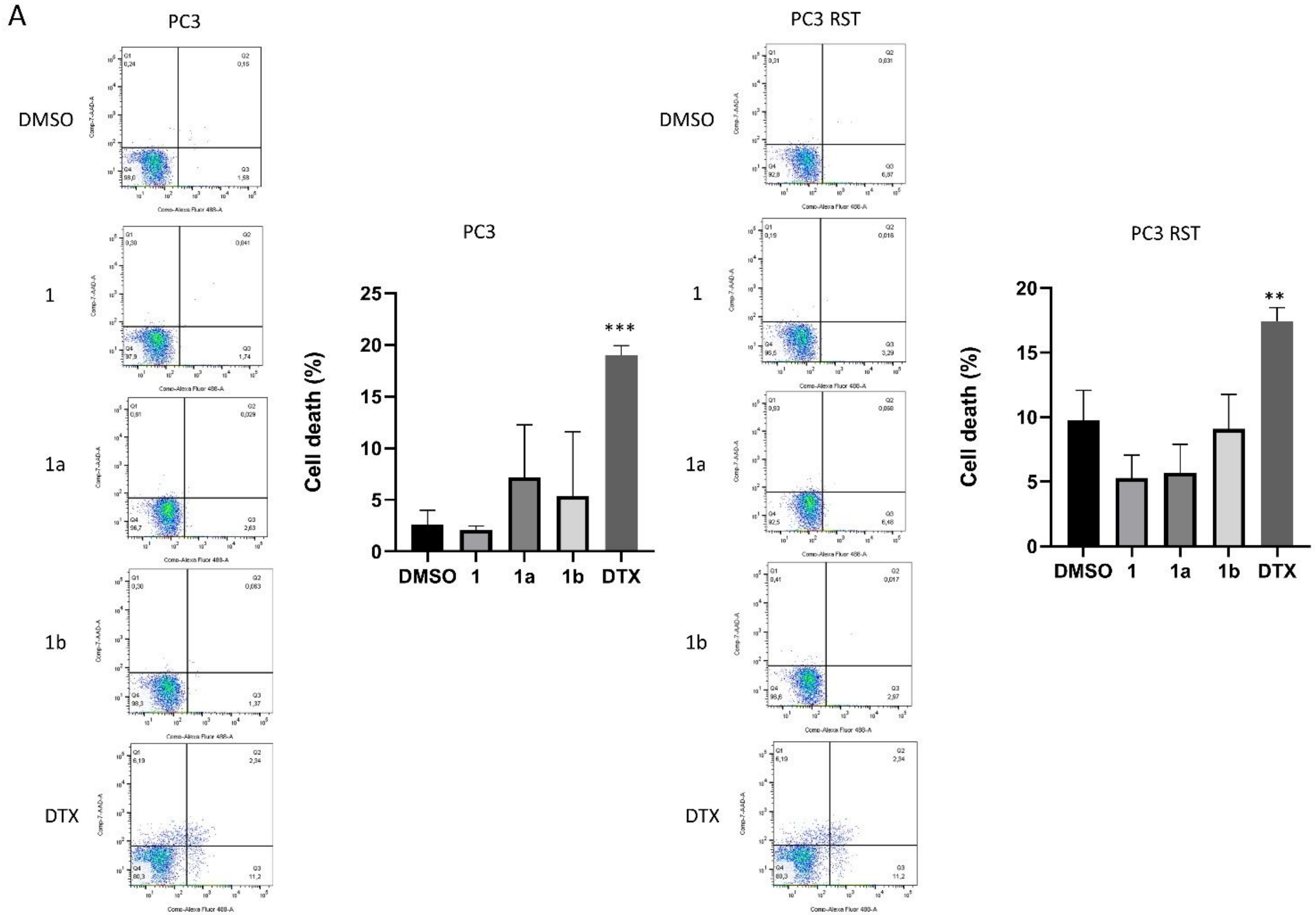

Prostate cancer cell lines, PC3 and PC3 RST (A); DU145 and DU145 RST (B) were treated with the IC50 of compounds, docetaxel (DTX) or vehicle (DMSO) for 72 hours, and an Annexin-V-FITC/7-AAD assay was performed. Asterisks indicate statistical significance compared with the control: *$p < 0.05$, **$p < 0.01$, ***$p < 0.001$.

B

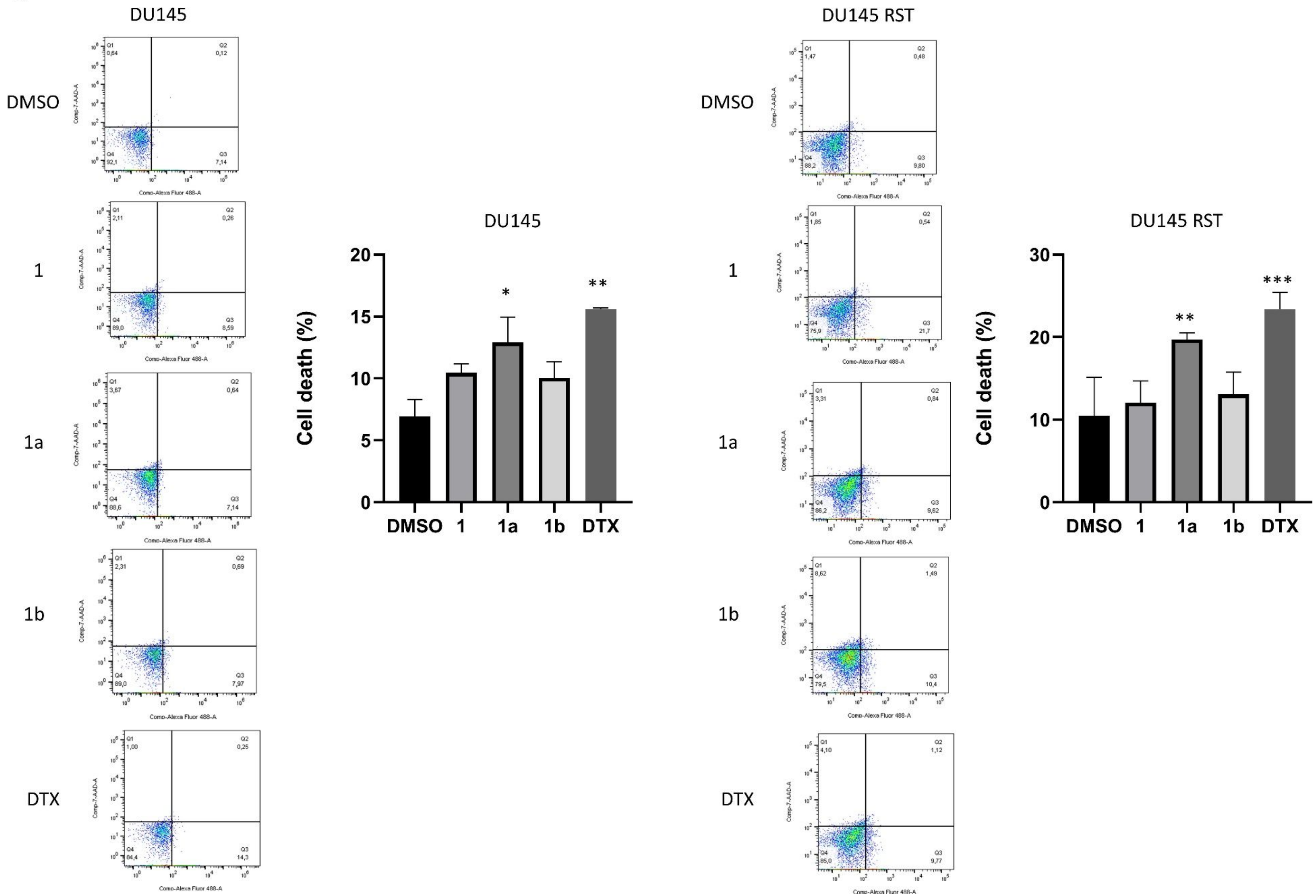

DU145
DU145 RST
DMSO
1
1a
1b
DTX
Comp-7-AAD-A
Comp-Alexa Fluor 488-A
Q1
Q2
Q3
Q4
DU145
Cell death (%)
DMSO
1
1a
1b
DTX
*
**
DU145 RST
Cell death (%)
**
***

Supplementary Figure S4. Raw and uncropped Western blot images corresponding to Figure 9.

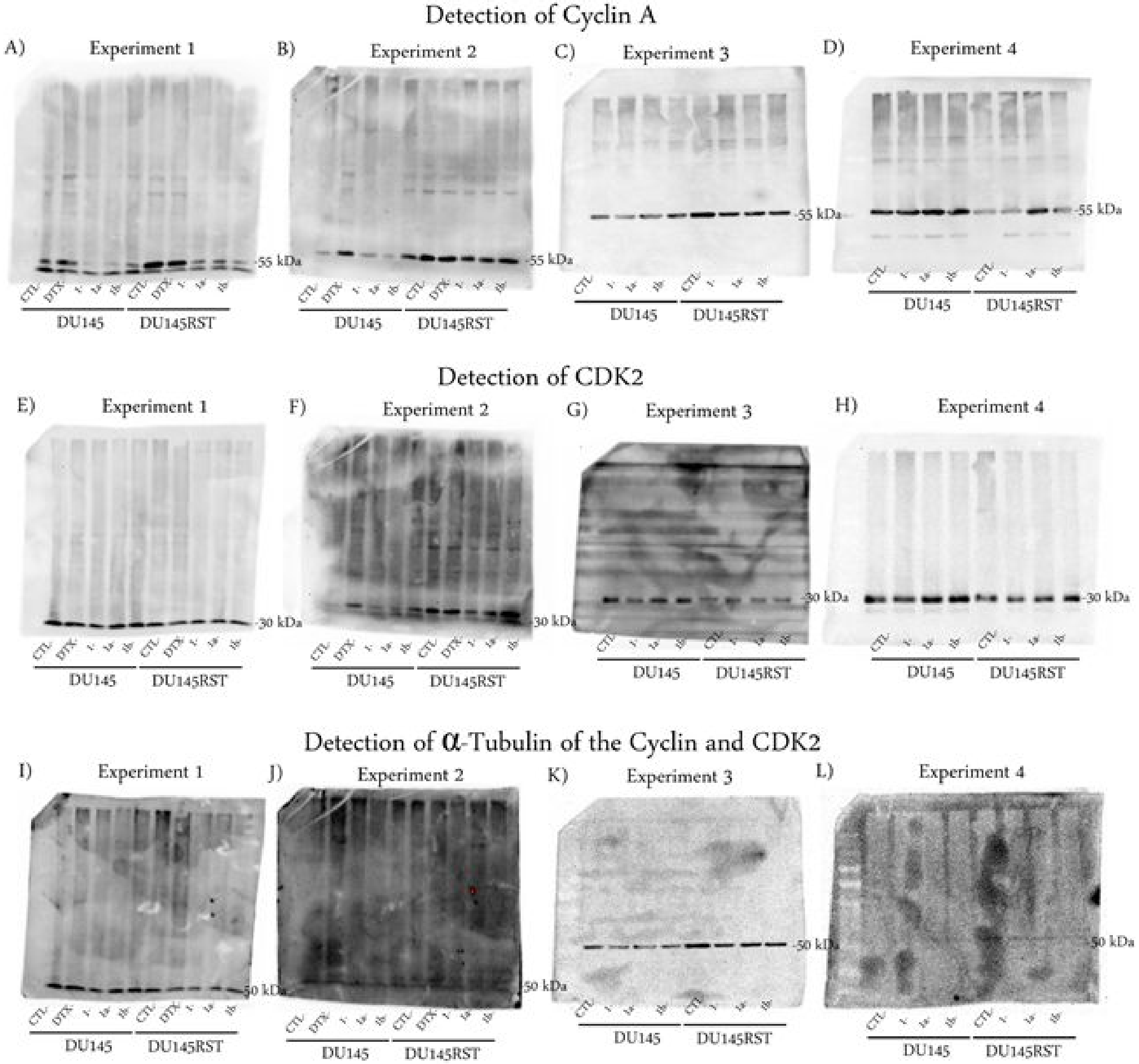

Raw and uncropped membrane images from four independent experiments assessing Cyclin A2 (~55 kDa), CDK2 (~30 kDa), and α-tubulin (~50 kDa) in DU145 and DU145RST cells after 48 h treatment with control (CTL), docetaxel (DTX), compound 1, compound 1a, or compound 1b at their respective IC_{50} concentrations. (A–D) Cyclin A2; (E–H) CDK2; and (I–L) the corresponding α-tubulin loading controls. For each experiment, lanes are ordered as CTL, DTX, 1, 1a, and 1b for DU145, followed by the same sequence for DU145RST. The cropped representative blots and densitometric quantifications are presented in Figure 9.

Supplementary Figure S5A. Centre-of-mass distance between each ligand and its respective binding site along the 100 ns MD simulation.

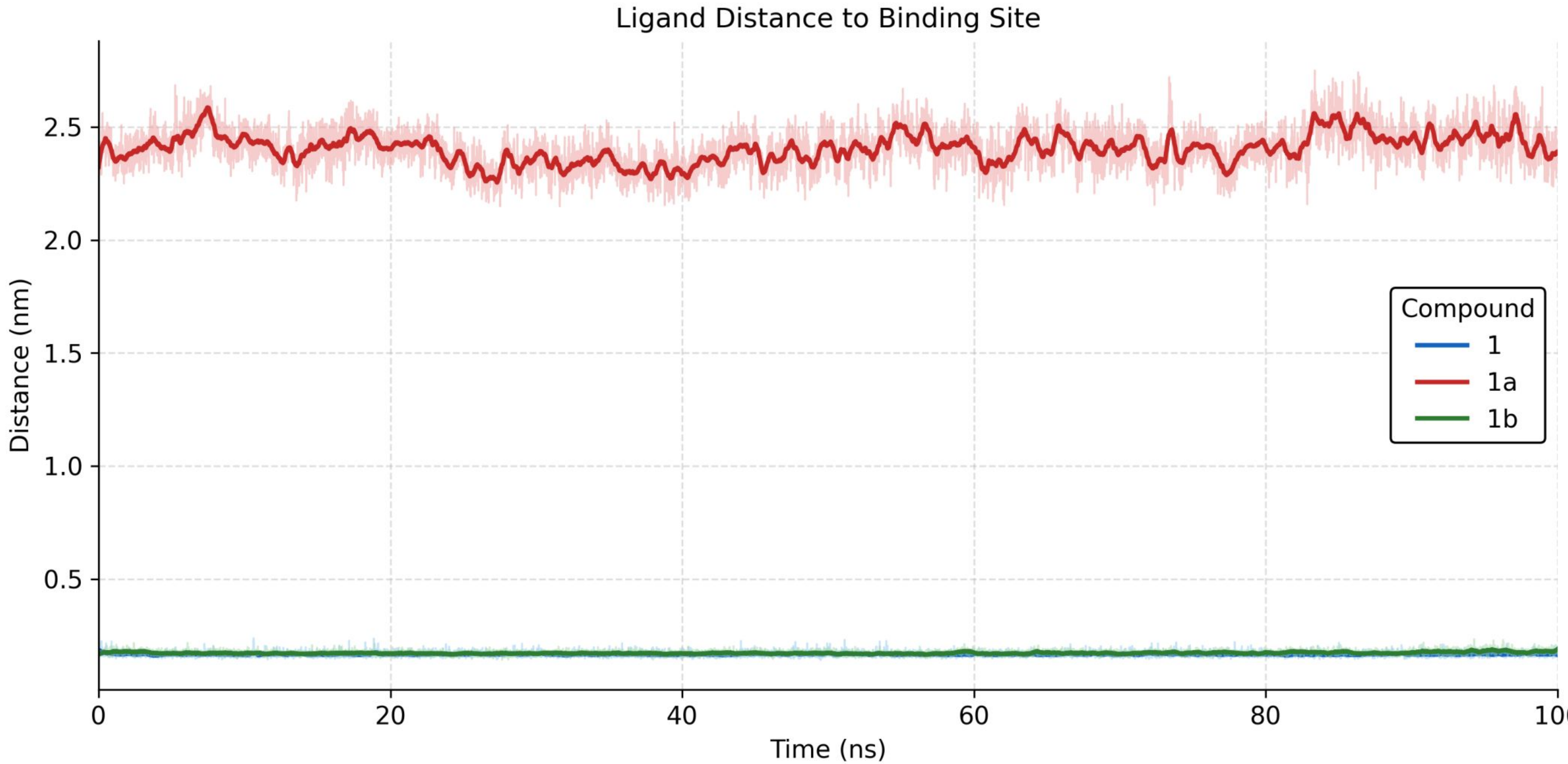

Ligand distance to binding site calculated as the centre-of-mass distance between each compound and the centroid of its respective docking pocket along the 100 ns trajectory. Compounds 1 (blue) and 1b (green) maintained stable distances of approximately 0.20 nm within the ATP-binding site throughout the entire simulation, confirming complete retention in the catalytic pocket. Compound 1a (red) was simulated at the CDK2/Cyclin A2 dimerization interface, exhibiting stable distances of approximately 2.4 nm relative to the ATP-site reference centroid, consistent with its distinct binding location at the activation interface.

Supplementary Figure S5B. Temporal contact map — Compound 1 (≤ 4.0 Å).

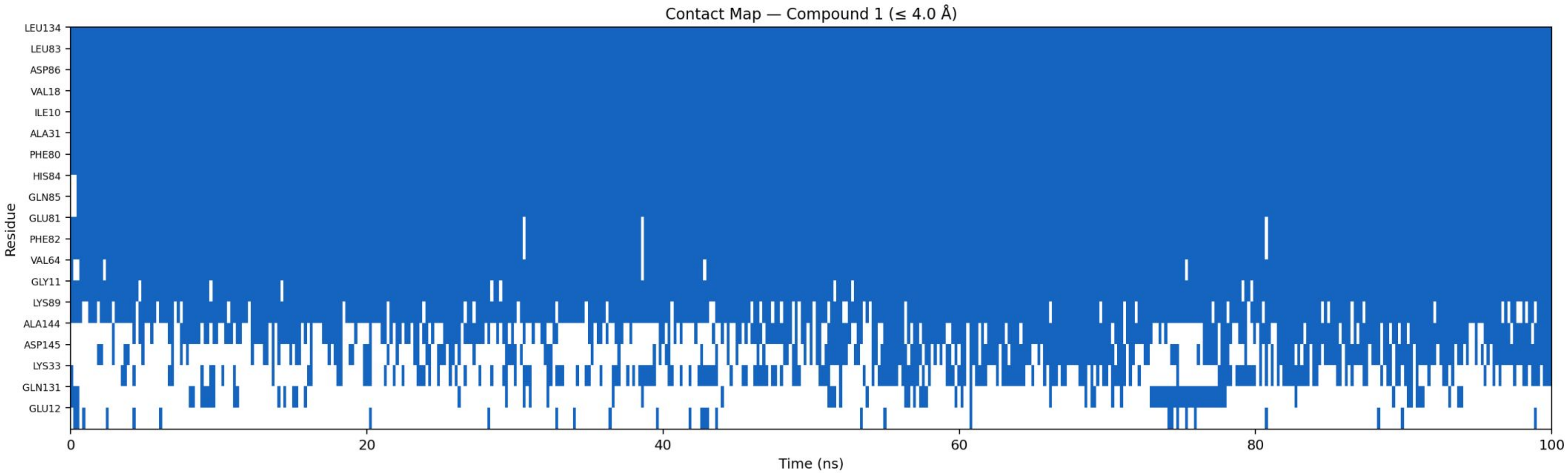

Residue-resolved temporal contact map for compound 1 at the CDK2 ATP-binding site over the 100 ns simulation. Blue bars indicate frames in which the ligand is within 4.0 Å of each labelled residue. The conserved catalytic core residues (LEU134, LEU83, ASP86, VAL18, ILE10, ALA31, PHE80, HIS84, GLN85, GLU81, PHE82) maintain near-continuous contacts throughout the trajectory. Flexible contacts are observed for peripheral residues including ALA144, ASP145, LYS89, LYS33, and GLN131.

Supplementary Figure S5C. Temporal contact map — Compound 1a (≤ 4.0 Å).

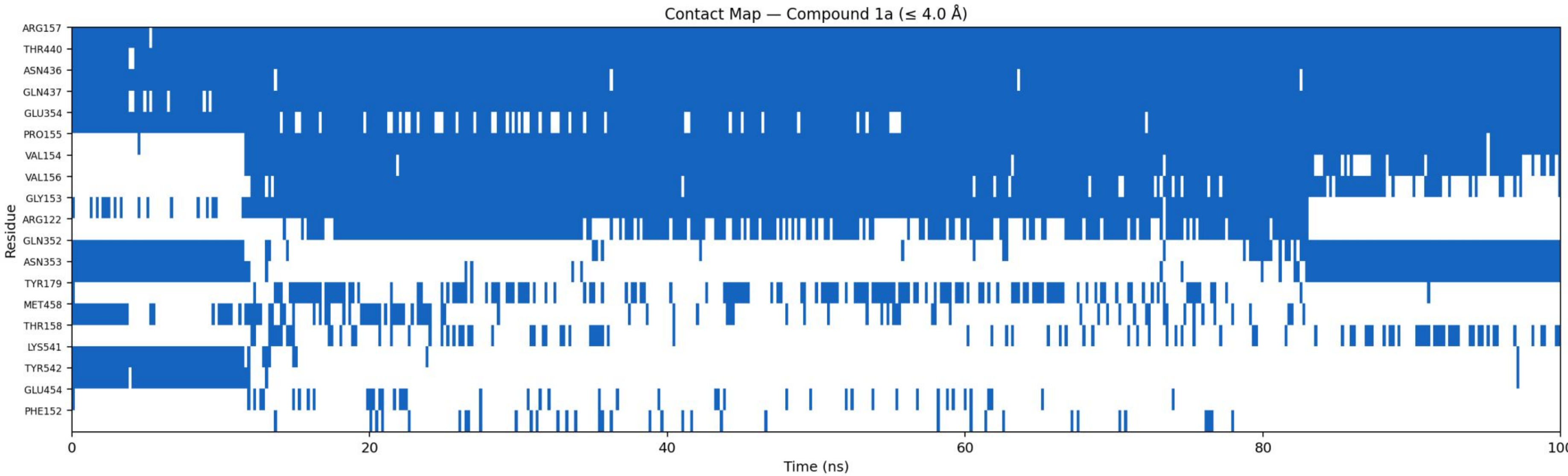

Residue-resolved temporal contact map for compound 1a at the CDK2/Cyclin A2 dimerization interface over the 100 ns simulation. Blue bars indicate frames in which the ligand is within 4.0 Å of each labelled residue. The contact profile is entirely distinct from those of compounds 1 and 1b, engaging exclusively interface residues including ARG157, THR440, ASN436, GLN437, GLU354, PRO155, VAL154, VAL156, GLY153, ARG122, GLN352, ASN353, TYR179, MET458, THR158, LYS541, TYR542, GLU454, and PHE152. The intermittent and dynamic contact pattern reflects the greater conformational flexibility of the open dimerization interface compared to the enclosed ATP pocket.

Supplementary Figure S5D. Temporal contact map — Compound 1b (≤ 4.0 Å).

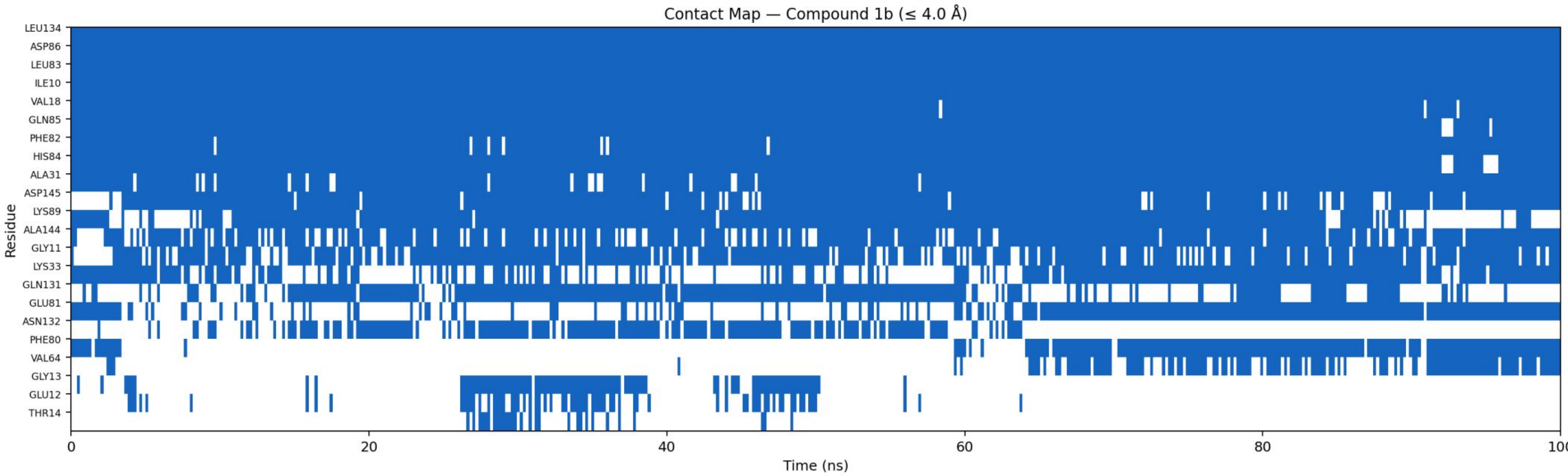

Residue-resolved temporal contact map for compound 1b at the CDK2 ATP-binding site over the 100 ns simulation. Blue bars indicate frames in which the ligand is within 4.0 Å of each labelled residue. Compared to compound 1, the removal of the C-7 hydroxyl group results in increased accessibility of the hinge region residues GLN131, ASN132, GLU12, and THR14, which exhibit sustained contacts particularly after 60 ns, consistent with the progressive pose convergence observed in the ligand RMSD analysis. The conserved catalytic core contact pattern (LEU134, LEU83, ASP86, VAL18, ILE10, ALA31, PHE82, HIS84, GLN85, GLU81, PHE80) is maintained with high frequency throughout the entire trajectory.

Supplementary Figure S6. Differential dynamic cross-correlation maps (ΔDCCM) relative to compound 1.

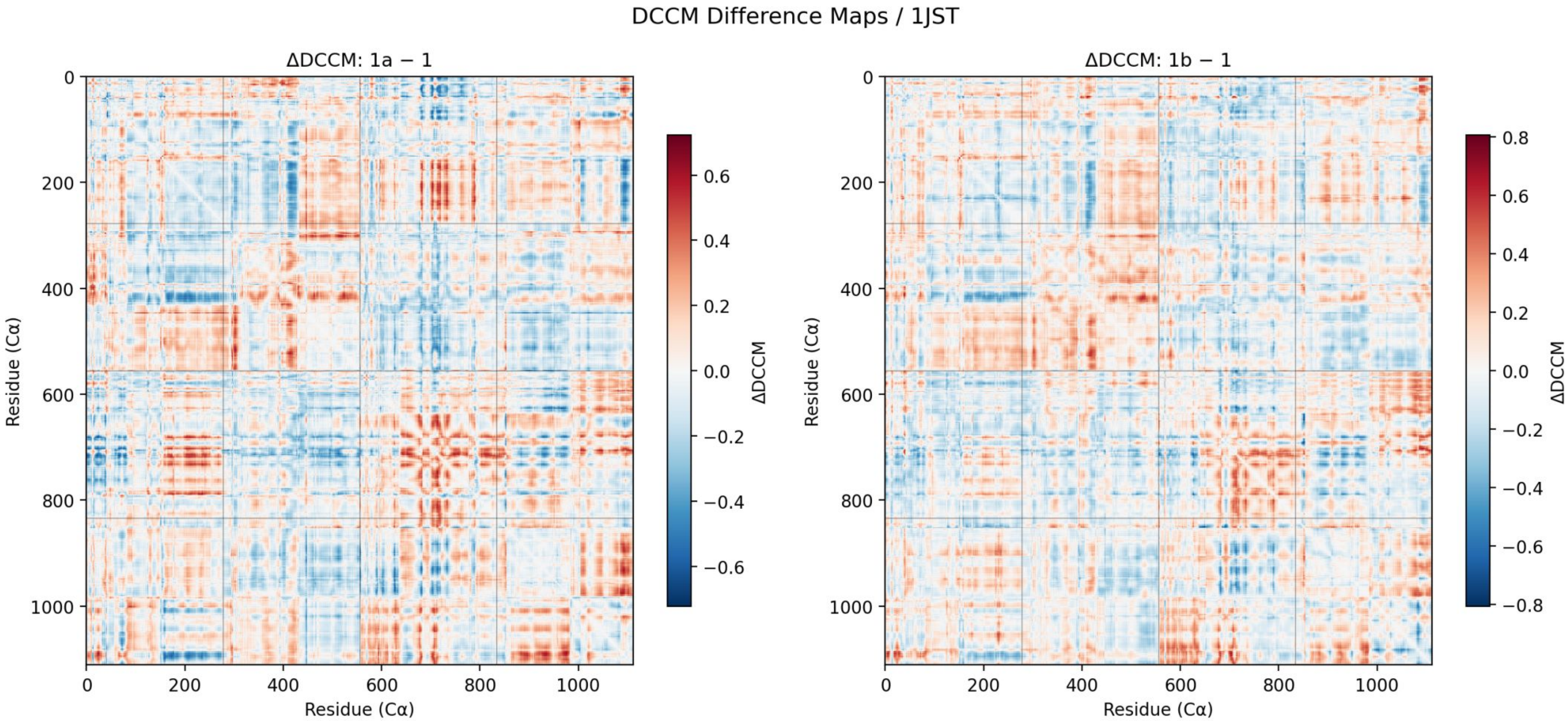

Differential dynamic cross-correlation matrices computed as ΔDCCM = DCCM(compound X) − DCCM(compound 1), where positive values (red) indicate enhanced correlated motions and negative values (blue) indicate reduced correlations relative to the reference system (compound 1). Left panel: ΔDCCM(1a − 1); right panel: ΔDCCM(1b − 1). The DCCM was calculated from Cα atom displacements over the full 100 ns trajectory for the CDK2/Cyclin A2 complex (PDB: 1JST). Gridlines delineate the boundaries between the four protein chains of the simulated tetramer. Compound 1a induces widespread increases in inter-domain and inter-subunit correlated motions (red blocks at CDK2–Cyclin A2 domain

boundaries), consistent with an interfacial stabilization mechanism that reorganizes collective dynamics across the dimer. Compound 1b produces a predominantly negative differential (blue regions), reflecting dampened correlations between CDK2 and Cyclin A2 residue blocks, particularly between chains A–B and C–D, which supports the allosteric perturbation originating from the modified C-7 position of the narciclasine scaffold.

Supplementary Table S1: Complete data, including the list of predicted targets per compound, fit scores, normalized fit scores for compounds 1, 1a and 1b

PDB ID	Target Name	Number of Features	Fit Score	Normalized Fit Score	z'-score
		**1**			
**1QZY**	Methionine aminopeptidase 2	9	4.43	0.4922	3.43698
**3CPB**	Vascular endothelial growth factor receptor 2	7	4.647	0.6638	3.23195
**1D8F**	Stromelysin-1	7	4.372	0.6246	3.17537
**2BTS**	Cell division protein kinase 2	6	4.586	0.7643	3.10001
**2RG6**	Mitogen-activated protein kinase 14	9	4.622	0.5136	2.86974
**2I3H**	Baculoviral IAP repeat-containing protein 7	8	4.196	0.5245	2.7386
**1DIC**	Complement factor D	4	3.843	0.9607	2.39386
**1G1D**	Carbonic anhydrase 2	5	4.138	0.8275	2.39237
**2HOG**	Serine/threonine-protein kinase Chk1	6	3.903	0.6506	2.25251
**1EF3**	Aldose reductase	6	4.109	0.6848	2.24822
**2OO8**	Angiopoietin-1 receptor	8	4.606	0.5757	2.22017
**1OIU**	Cyclin-A2	6	3.833	0.6388	2.20938
**3EY4**	Corticosteroid 11-beta-dehydrogenase isozyme 1	5	3.876	0.7751	2.17459

PDB ID	Target Name	Number of Features	Fit Score	Normalized Fit Score	z'-score
		**1**			
**2CNH**	Tyrosine-protein phosphatase non-receptor type 1	7	3.866	0.5522	2.14659
**1W7G**	Prothrombin	6	3.747	0.6245	2.11018
**3DNG**	Neutrophil collagenase	7	3.814	0.5449	2.06304
**2A8H**	ADAM 17	6	3.754	0.6256	2.00712
**1K3Y**	Glutathione S-transferase A1	9	3.763	0.4181	1.94083
**1UZF**	Angiotensin-converting enzyme	7	4.701	0.6716	1.89603
**1OSF**	Heat shock protein HSP 90-alpha	12	4.832	0.4027	1.87781
**2WI1**	NONE	4	3.677	0.9193	1.84595
**2RKU**	Serine/threonine-protein kinase PLK1	4	3.621	0.9053	1.81742
**2I6A**	Adenosine kinase	5	3.657	0.7314	1.81692
**1XIL**	Superoxide dismutase [Mn], mitochondrial	5	3.624	0.7249	1.79922
**1W22**	Histone deacetylase 8	6	4.086	0.681	1.78301
**1DQ9**	3-hydroxy-3-methylglutaryl-coenzyme A reductase	9	3.621	0.4023	1.77386
**1Q41**	Glycogen synthase kinase-3 beta	5	3.6	0.72	1.77144
**1I00**	Thymidylate synthase	7	3.814	0.5448	1.77047
**2NN7**	Carbonic anhydrase 1	4	3.738	0.9345	1.76811
**1OG5**	Cytochrome P450 2C9	6	3.842	0.6403	1.72934
**2OAH**	Beta-secretase 1	7	3.706	0.5294	1.6943

PDB ID	Target Name	Number of Features	Fit Score	Normalized Fit Score	z'-score
		**1**			
**1MEN**	Trifunctional purine biosynthetic protein adenosine-3	8	4.122	0.5152	1.69165
**1TFQ**	Baculoviral IAP repeat-containing protein 4	7	3.786	0.5409	1.66436
**1QCF**	Tyrosine-protein kinase HCK	7	4.19	0.5986	1.6346
**1UTT**	Macrophage metalloelastase	5	3.615	0.7231	1.63007
**1SVH**	cAMP-dependent protein kinase, alpha-catalytic subunit	7	3.756	0.5366	1.60628
**2GPQ**	Eukaryotic translation initiation factor 4E	8	3.568	0.446	1.59721
**2AAX**	Mineralocorticoid receptor	6	3.562	0.5936	1.57437
**1ONQ**	T-cell surface glycoprotein CD1a	6	3.479	0.5798	1.54008
**1TOU**	Fatty acid-binding protein, adipocyte	6	3.458	0.5764	1.51791
**1M2Z**	Glucocorticoid receptor	8	3.699	0.4624	1.50992
**1M48**	Interleukin-2	7	3.873	0.5534	1.50851
**1DIA**	C-1-tetrahydrofolate synthase, cytoplasmic	6	3.494	0.5824	1.49449
**1FZV**	Placenta growth factor	5	3.512	0.7024	1.44669
**1KMS**	Dihydrofolate reductase	7	3.538	0.5054	1.43966
**2P3T**	Coagulation factor X	6	3.626	0.6044	1.43031
**1HS6**	Leukotriene A-4 hydrolase	9	3.981	0.4423	1.42916
**1KNU**	Peroxisome proliferator-activated receptor gamma	6	3.533	0.5888	1.42274
**2VWV**	Ephrin type-B receptor 4	5	3.536	0.7072	1.40706

PDB ID	Target Name	Number of Features	Fit Score	Normalized Fit Score	z'-score
		**1**			
**1Q4N**	Alpha-amylase 1	6	3.507	0.5844	1.39278
**1CM0**	Histone acetyltransferase PCAF	9	3.648	0.4053	1.38906
**2PE0**	3-phosphoinositide-dependent protein kinase 1	4	3.451	0.8628	1.38083
**1ZOM**	Coagulation factor XI	8	3.456	0.432	1.36428
**1O4J**	Proto-oncogene tyrosine-protein kinase Src	5	3.617	0.7233	1.35178
**1RY8**	Aldo-keto reductase family 1 member C3	5	3.398	0.6796	1.34454
**1IRJ**	Protein S100-A9	7	3.675	0.525	1.32179
**1P62**	Deoxycytidine kinase	7	3.631	0.5188	1.28683
**2IL2**	Renin	8	3.517	0.4396	1.27785
**1NQC**	Cathepsin S	7	3.665	0.5236	1.24441
**2OG8**	Proto-oncogene tyrosine-protein kinase LCK	11	4.096	0.3724	1.22642
**1RWQ**	Dipeptidyl peptidase 4	7	3.728	0.5325	1.21952
**1OYN**	cAMP-specific 3,5-cyclic phosphodiesterase 4D	5	3.434	0.6869	1.18765
**1HOV**	72 kDa type IV collagenase	7	3.669	0.5241	1.18726
**2ZM3**	Insulin-like growth factor 1 receptor	6	3.579	0.5965	1.17681
**1EKV**	Branched-chain-amino-acid aminotransferase, mitochondrial	8	3.764	0.4705	1.1583
**1Q11**	Tyrosyl-tRNA synthetase, cytoplasmic	5	3.384	0.6769	1.15788
**1MX1**	Liver carboxylesterase 1	4	3.412	0.8531	1.14921

PDB ID	Target Name	Number of Features	Fit Score	Normalized Fit Score	z'-score
		**1**			
**1ND5**	Prostatic acid phosphatase	6	3.71	0.6184	1.13768
**2CAN**	Ornithine aminotransferase, mitochondrial	10	3.738	0.3738	1.13468
**13GS**	Glutathione S-transferase P	6	3.593	0.5988	1.12435
**2FS9**	Tryptase beta-2	8	3.386	0.4233	1.11821
**1QPF**	Peptidyl-prolyl cis-trans isomerase FKBP1A	8	3.592	0.449	1.09445
**1M9J**	Nitric oxide synthase, endothelial	4	3.396	0.849	1.08388
**1R7U**	Histo-blood group ABO system transferase	9	3.814	0.4238	1.07224
**2AUH**	Insulin receptor	11	4.208	0.3825	1.06305
**2B7A**	Tyrosine-protein kinase JAK2	7	3.779	0.5399	1.05423
**1VJ5**	Epoxide hydrolase 2	5	3.461	0.6921	1.03171
**1AGW**	Basic fibroblast growth factor receptor 1	4	3.305	0.8263	1.02146
**1PL6**	Sorbitol dehydrogenase	5	3.567	0.7134	1.0177
**1EQU**	Estradiol 17-beta-dehydrogenase 1	5	3.552	0.7104	1.01431
**1RO9**	cAMP-specific 3,5-cyclic phosphodiesterase 4B	6	3.553	0.5922	1.01018
**1OTH**	Ornithine carbamoyltransferase, mitochondrial	8	3.713	0.4641	1.00865
**1MKP**	Dual specificity protein phosphatase 6	4	3.275	0.8189	0.976366
**1SR7**	Progesterone receptor	8	3.654	0.4567	0.959941
**1MMT**	Phenylalanine-4-hydroxylase	8	3.527	0.4408	0.935441

PDB ID	Target Name	Number of Features	Fit Score	Normalized Fit Score	z'-score
		**1**			
**1ISJ**	ADP-ribosyl cyclase 2	7	3.388	0.484	0.931101
**1GKC**	Matrix metalloproteinase-9	8	3.743	0.4679	0.914651
**1BMQ**	Caspase-1	11	3.811	0.3464	0.914452
**1YZ3**	Phenylethanolamine N-methyltransferase	11	3.909	0.3554	0.908306
**2H44**	cGMP-specific 3,5-cyclic phosphodiesterase	7	3.264	0.4663	0.8917
**2BXV**	Dihydroorotate dehydrogenase, mitochondrial	7	3.657	0.5225	0.875119
**1LT8**	Betaine--homocysteine S-methyltransferase 1	8	3.569	0.4462	0.872462
**2YXJ**	Bcl-2-like protein 1	9	3.426	0.3807	0.859798
**2F4J**	Proto-oncogene tyrosine-protein kinase ABL1	8	3.552	0.4441	0.855584
**1UJ2**	Uridine-cytidine kinase 2	8	3.666	0.4583	0.839745
**1N6O**	Ras-related protein Rab-5A	9	3.656	0.4062	0.809131
**1PBK**	FK506-binding protein 3	11	3.43	0.3118	0.805615
**1TFG**	Transforming growth factor beta-2	9	3.77	0.4189	0.802055
**1KQU**	Phospholipase A2, membrane associated	8	3.835	0.4793	0.789946

		**1A**			
PDB ID	Target Name	Number of Features	Fit Score	Normalized Fit Score	z'-score
**2C6L**	Cell division protein kinase 2	5	4.236	0.8473	3.85651

PDB ID	Target Name	Number of Features	Fit Score	Normalized Fit Score	z'-score
**2BU5**	[Pyruvate dehydrogenase [lipoamide]] kinase isozyme 2, mitochondrial	6	4.4	0.7334	3.35768
**1LZI**	Histo-blood group ABO system transferase	7	3.866	0.5522	3.08073
**1J4I**	Peptidyl-prolyl cis-trans isomerase FKBP1A	7	4.058	0.5796	3.0512
**1Q41**	Glycogen synthase kinase-3 beta	5	3.779	0.7557	3.03795
**1XH7**	cAMP-dependent protein kinase catalytic subunit alpha	8	4.09	0.5113	3.01123
**1T67**	Histone deacetylase 8	6	4.087	0.6811	2.93099
**1BJ4**	Serine hydroxymethyltransferase, cytosolic	6	4.038	0.6731	2.87741
**1S2A**	Aldo-keto reductase family 1 member C3	7	4.361	0.623	2.86529
**1YM2**	Beta-secretase 1	13	4.808	0.3698	2.79676
**2ZB0**	Mitogen-activated protein kinase 14	8	4.462	0.5578	2.63417
**2C6T**	Cyclin-A2	7	4.314	0.6163	2.60363
**1RO9**	cAMP-specific 3,5-cyclic phosphodiesterase 4B	6	3.834	0.6389	2.58161
**3D3E**	Corticosteroid 11-beta-dehydrogenase isozyme 1	6	3.891	0.6486	2.57582
**3C4C**	B-Raf proto-oncogene serine/threonine-protein kinase	8	4.163	0.5204	2.40845
**1Q6P**	Tyrosine-protein phosphatase non-receptor type 1	9	4.24	0.4711	2.34604
**1PMU**	Mitogen-activated protein kinase 10	4	3.543	0.8856	2.32523

PDB ID	Target Name	Number of Features	Fit Score	Normalized Fit Score	z'-score
**2B7A**	Tyrosine-protein kinase JAK2	7	4.025	0.575	2.29112
**1P93**	Glucocorticoid receptor	9	3.95	0.4389	2.22803
**2BX8**	Serum albumin	5	3.744	0.7489	2.15384
**1PQC**	Oxysterols receptor LXR-beta	6	3.691	0.6152	2.15034
**3DNG**	Neutrophil collagenase	7	3.535	0.505	2.10703
**1BNV**	Carbonic anhydrase 2	7	3.78	0.54	2.04021
**2PJL**	Steroid hormone receptor ERR1	5	3.536	0.7072	2.02656
**2WI5**	NONE	6	3.491	0.5818	1.99828
**2BUB**	Dipeptidyl peptidase 4	5	3.481	0.6963	1.92541
**1DIC**	Complement factor D	4	3.365	0.8413	1.92324
**1C9H**	Peptidyl-prolyl cis-trans isomerase FKBP1B	9	3.443	0.3826	1.92254
**1HRK**	Ferrochelatase, mitochondrial	8	4.008	0.5011	1.849
**1XOR**	cAMP-specific 3,5-cyclic phosphodiesterase 4D	5	3.455	0.6909	1.78554
**2BYH**	Heat shock protein HSP 90-alpha	5	3.299	0.6599	1.74191
**1UHL**	Oxysterols receptor LXR-alpha	6	3.589	0.5982	1.74077
**3BEJ**	Bile acid receptor	7	3.596	0.5137	1.73878

PDB ID	Target Name	Number of Features	Fit Score	Normalized Fit Score	z'-score
**1LE6**	Group 10 secretory phospholipase A2	4	3.368	0.8421	1.73312
**2G22**	Renin	5	3.29	0.658	1.70179
**1ZPB**	Coagulation factor XI	8	3.546	0.4433	1.68012
**1HMS**	Fatty acid-binding protein, heart	8	4.256	0.532	1.67478
**1Q4N**	Alpha-amylase 1	6	3.369	0.5615	1.67336
**2I47**	ADAM 17	7	3.363	0.4804	1.63417
**1D3H**	Dihydroorotate dehydrogenase, mitochondrial	6	3.487	0.5812	1.63032
**1D8F**	Stromelysin-1	7	3.411	0.4873	1.58097
**1QCF**	Tyrosine-protein kinase HCK	7	3.804	0.5434	1.58047
**1YPE**	Prothrombin	5	3.355	0.6711	1.57996
**3B65**	Androgen receptor	6	3.388	0.5647	1.56039
**1S95**	Serine/threonine-protein phosphatase 5	4	3.283	0.8208	1.52836
**2PE0**	3-phosphoinositide-dependent protein kinase 1	4	3.267	0.8167	1.52121
**2BIK**	Proto-oncogene serine/threonine-protein kinase Pim-1	7	3.568	0.5098	1.51701
**1KNU**	Peroxisome proliferator-activated receptor gamma	6	3.303	0.5505	1.50077
**1ISJ**	ADP-ribosyl cyclase 2	7	3.321	0.4744	1.47313

PDB ID	Target Name	Number of Features	Fit Score	Normalized Fit Score	z'-score
**2AAX**	Mineralocorticoid receptor	6	3.26	0.5434	1.47017
**1EGC**	Medium-chain specific acyl-CoA dehydrogenase, mitochondrial	7	3.226	0.4609	1.46008
**2P1T**	Retinoic acid receptor RXR-alpha	7	3.388	0.484	1.45832
**1Z6E**	Coagulation factor X	6	3.297	0.5494	1.44316
**1I7G**	Peroxisome proliferator-activated receptor alpha	6	3.43	0.5717	1.43651
**1PIC**	Phosphatidylinositol 3-kinase regulatory subunit alpha	9	3.44	0.3822	1.41857
**1YVJ**	Tyrosine-protein kinase JAK3	6	3.489	0.5815	1.41316
**3BE2**	Vascular endothelial growth factor receptor 2	8	3.738	0.4673	1.41039
**2RKU**	Serine/threonine-protein kinase PLK1	4	3.182	0.7955	1.406
**1IZ2**	Alpha-1-antitrypsin	6	3.377	0.5629	1.40442
**1S9J**	Dual specificity mitogen-activated protein kinase kinase 1	9	4.274	0.4749	1.39348
**1Q20**	Sulfotransferase family cytosolic 2B member 1	9	3.656	0.4062	1.38513
**1DXO**	NAD(P)H dehydrogenase [quinone] 1	6	3.436	0.5726	1.36252
**1ORE**	Adenine phosphoribosyltransferase	7	3.479	0.4971	1.32699
**1S19**	Vitamin D3 receptor	8	3.495	0.4369	1.32076
**1X98**	Aldose reductase	6	3.368	0.5613	1.29333

PDB ID	Target Name	Number of Features	Fit Score	Normalized Fit Score	z'-score
**3BBT**	Receptor tyrosine-protein kinase erbB-4	6	3.38	0.5634	1.27129
**1SVH**	cAMP-dependent protein kinase, alpha-catalytic subunit	7	3.325	0.475	1.25601
**1PW6**	Interleukin-2	8	3.454	0.4318	1.24491
**2Q70**	Estrogen receptor	5	3.291	0.6581	1.2423
**2FGI**	Basic fibroblast growth factor receptor 1	8	3.873	0.4842	1.23235
**2C3K**	Serine/threonine-protein kinase Chk1	6	3.288	0.548	1.23041
**1IMX**	Insulin-like growth factor IA	7	3.492	0.4989	1.20245
**1XUD**	Collagenase 3	7	3.247	0.4639	1.17303
**1XO2**	Cell division protein kinase 6	5	3.165	0.6329	1.17017
**1O43**	Proto-oncogene tyrosine-protein kinase Src	9	3.47	0.3856	1.15479
**1QPD**	Proto-oncogene tyrosine-protein kinase LCK	6	3.45	0.575	1.15024
**1H6G**	Catenin alpha-1	5	3.202	0.6404	1.1458
**1I00**	Thymidylate synthase	7	3.275	0.4679	1.14377
**3F5P**	Insulin-like growth factor 1 receptor	4	3.099	0.7747	1.13411
**1ROS**	Macrophage metalloelastase	10	3.51	0.351	1.13322
**2OJJ**	Mitogen-activated protein kinase 1	7	3.4	0.4858	1.12145

PDB ID	Target Name	Number of Features	Fit Score	Normalized Fit Score	z'-score
**1NXK**	MAP kinase-activated protein kinase 2	7	3.456	0.4937	1.1099
**1TFQ**	Baculoviral IAP repeat-containing protein 4	7	3.262	0.466	1.10776
**1R1I**	Neprilysin	8	3.479	0.4349	1.09725
**1R0P**	Hepatocyte growth factor receptor	6	3.437	0.5729	1.08416
**1U59**	Tyrosine-protein kinase ZAP-70	7	3.428	0.4897	1.04283
**1SM2**	Tyrosine-protein kinase ITK/TSK	6	3.398	0.5664	1.0428
**1MX1**	Liver carboxylesterase 1	4	3.134	0.7836	1.03904
**1NN0**	Thymidylate kinase	8	3.523	0.4403	1.03498
**1XKK**	Epidermal growth factor receptor	9	3.287	0.3652	1.01855
**1N83**	Nuclear receptor ROR-alpha	4	3.114	0.7784	1.0136
**1P62**	Deoxycytidine kinase	7	3.26	0.4657	1.00927
**1EXX**	Retinoic acid receptor gamma	9	3.372	0.3747	0.996143
**1QYW**	Estradiol 17-beta-dehydrogenase 1	5	3.233	0.6466	0.943596
**1E5A**	Transthyretin	4	3.079	0.7697	0.899913
**3B2R**	cGMP-specific 3,5-cyclic phosphodiesterase	6	3.162	0.5269	0.897804
**2BK3**	Amine oxidase [flavin-containing] B	7	3.858	0.5512	0.886228

PDB ID	Target Name	Number of Features	Fit Score	Normalized Fit Score	z'-score
**1LHN**	Sex hormone-binding globulin	6	3.44	0.5734	0.885596
**2J4A**	Thyroid hormone receptor beta	6	3.195	0.5326	0.880483

1B					
PDB ID	Target Name	Number of Features	Fit Score	Normalized Fit Score	z'-score
**1Y2B**	cAMP-specific 3,5-cyclic phosphodiesterase 4D	5	4.455	0.8911	3.83308
**1G45**	Carbonic anhydrase 2	5	4.211	0.8422	3.52638
**1DWD**	Prothrombin	7	4.161	0.5944	2.89275
**1IMX**	Insulin-like growth factor IA	7	4.324	0.6177	2.87355
**2JDS**	cAMP-dependent protein kinase catalytic subunit alpha	7	4.071	0.5816	2.78877
**1SZ7**	Trafficking protein particle complex subunit 3	6	4.337	0.7229	2.75515
**3CE3**	Hepatocyte growth factor receptor	8	4.309	0.5386	2.73633
**3GZ9**	NONE	9	4.384	0.4871	2.48121
**1HA2**	Serum albumin	6	4.159	0.6931	2.45896
**2I4Q**	Renin	7	3.816	0.5451	2.40404
**1ZZL**	Mitogen-activated protein kinase 14	7	4.224	0.6034	2.37718
**1Q11**	Tyrosyl-tRNA synthetase, cytoplasmic	5	3.697	0.7393	2.27941

PDB ID	Target Name	Number of Features	Fit Score	Normalized Fit Score	z'-score
**1PW6**	Interleukin-2	8	4.001	0.5001	2.25249
**2FJP**	Dipeptidyl peptidase 4	6	3.663	0.6104	2.24449
**1UEJ**	Uridine-cytidine kinase 2	6	3.656	0.6094	2.21764
**2G8N**	Phenylethanolamine N-methyltransferase	5	3.729	0.7457	2.20634
**1JK7**	Serine/threonine-protein phosphatase PP1-gamma catalytic subunit	9	4.148	0.4608	2.19562
**3EY4**	Corticosteroid 11-beta-dehydrogenase isozyme 1	5	3.695	0.739	2.12665
**1QCF**	Tyrosine-protein kinase HCK	7	4.19	0.5985	2.10406
**2FZZ**	Coagulation factor X	5	3.621	0.7241	2.10096
**2QXS**	Estrogen receptor	6	3.844	0.6407	2.09208
**1DI8**	Cell division protein kinase 2	6	3.731	0.6218	2.09036
**1OG5**	Cytochrome P450 2C9	6	3.808	0.6347	2.05441
**1W6K**	Lanosterol synthase	7	4.071	0.5816	2.04663
**1XLS**	Retinoic acid receptor RXR-alpha	6	3.694	0.6157	2.04379
**1LZJ**	B transferase	6	3.527	0.5878	2.01034
**1R5G**	Methionine aminopeptidase 2	9	3.782	0.4202	1.97998
**3CCW**	3-hydroxy-3-methylglutaryl-coenzyme A reductase	8	3.739	0.4674	1.95537

PDB ID	Target Name	Number of Features	Fit Score	Normalized Fit Score	z'-score
**1DHT**	Estradiol 17-beta-dehydrogenase 1	6	4.05	0.675	1.92306
**2P4I**	Angiopoietin-1 receptor	9	4.345	0.4828	1.91765
**1XS7**	Beta-secretase 1	11	4.298	0.3907	1.90534
**2I0G**	Estrogen receptor beta	4	3.66	0.9149	1.88259
**1FZV**	Placenta growth factor	5	3.556	0.7113	1.87174
**1IZ2**	Alpha-1-antitrypsin	6	3.695	0.6158	1.83811
**2C3K**	Serine/threonine-protein kinase Chk1	6	3.681	0.6135	1.83699
**1YRS**	Kinesin-like protein KIF11	5	3.554	0.7108	1.83633
**1NQ2**	Thyroid hormone receptor beta	7	3.854	0.5506	1.81991
**1R7T**	Histo-blood group ABO system transferase	6	3.582	0.597	1.80633
**1KMS**	Dihydrofolate reductase	7	3.534	0.5049	1.80214
**1X98**	Aldose reductase	6	3.682	0.6136	1.79339
**1AU3**	Cathepsin K	7	3.505	0.5008	1.79003
**1HE3**	Flavin reductase	6	3.668	0.6114	1.77004
**3BBT**	Receptor tyrosine-protein kinase erbB-4	6	3.71	0.6183	1.7635
**1XUC**	Collagenase 3	5	3.428	0.6855	1.73747

PDB ID	Target Name	Number of Features	Fit Score	Normalized Fit Score	z'-score
**1FM6**	Peroxisome proliferator-activated receptor gamma	4	3.503	0.8758	1.72949
**2RL5**	Vascular endothelial growth factor receptor 2	5	3.609	0.7219	1.72132
**1XKK**	Epidermal growth factor receptor	9	3.709	0.4121	1.70021
**1H1B**	Leukocyte elastase	6	3.63	0.605	1.6912
**1U4M**	C-C motif chemokine 5	8	3.442	0.4302	1.67283
**1P62**	Deoxycytidine kinase	7	3.644	0.5205	1.66146
**2F4J**	Proto-oncogene tyrosine-protein kinase ABL1	8	3.807	0.4759	1.65342
**2CNG**	Tyrosine-protein phosphatase non-receptor type 1	8	3.652	0.4565	1.64084
**1J78**	Vitamin D-binding protein	4	3.698	0.9246	1.58969
**1D5J**	Stromelysin-1	8	3.782	0.4728	1.58561
**1MMB**	Neutrophil collagenase	8	3.791	0.4739	1.58378
**1GSE**	Glutathione S-transferase A1	6	3.794	0.6323	1.58369
**1S8C**	Heme oxygenase 1	8	3.514	0.4392	1.57956
**3CLD**	Glucocorticoid receptor	8	3.703	0.4628	1.5771
**1Y2J**	cAMP-specific 3,5-cyclic phosphodiesterase 4B	7	3.76	0.5371	1.5741
**1RW8**	TGF-beta receptor type-1	6	3.924	0.654	1.57085

PDB ID	Target Name	Number of Features	Fit Score	Normalized Fit Score	z'-score
**2JDR**	Glycogen synthase kinase-3 beta	7	3.593	0.5134	1.56257
**2FV5**	ADAM 17	6	3.583	0.5972	1.55091
**1FKG**	Peptidyl-prolyl cis-trans isomerase FKBP1A	7	3.724	0.532	1.51546
**1LE6**	Group 10 secretory phospholipase A2	4	3.411	0.8528	1.48759
**1O6U**	SEC14-like protein 2	5	3.772	0.7544	1.47915
**2QG2**	Heat shock protein HSP 90-alpha	5	3.457	0.6915	1.47104
**1SKX**	Nuclear receptor subfamily 1 group I member 2	10	4.465	0.4465	1.45676
**1Z95**	Androgen receptor	8	3.79	0.4738	1.43707
**1L7X**	Glycogen phosphorylase, liver form	7	3.481	0.4973	1.41964
**1R5L**	Alpha-tocopherol transfer protein	12	4.755	0.3963	1.39689
**1M6D**	Cathepsin F	7	3.509	0.5013	1.39467
**1L8J**	Endothelial protein C receptor	13	4.009	0.3084	1.38338
**2E2R**	Estrogen-related receptor gamma	5	3.477	0.6954	1.35188
**1N83**	Nuclear receptor ROR-alpha	4	3.365	0.8414	1.33819
**1IH0**	Troponin C, slow skeletal and cardiac muscles	4	3.326	0.8314	1.3117
**1M51**	Phosphoenolpyruvate carboxykinase, cytosolic [GTP]	6	4.066	0.6777	1.29911

PDB ID	Target Name	Number of Features	Fit Score	Normalized Fit Score	z'-score
**1T67**	Histone deacetylase 8	6	3.573	0.5954	1.29572
**2AUH**	Insulin receptor	11	4.112	0.3738	1.28625
**1O4J**	Proto-oncogene tyrosine-protein kinase Src	5	3.471	0.6943	1.27985
**1NAV**	Thyroid hormone receptor alpha	7	3.804	0.5435	1.26532
**1WOK**	Poly [ADP-ribose] polymerase 1	5	3.44	0.6881	1.25219
**4LBD**	Retinoic acid receptor gamma	10	3.837	0.3837	1.24637
**1IVH**	Isovaleryl-CoA dehydrogenase, mitochondrial	12	3.591	0.2992	1.20623
**1PMN**	Mitogen-activated protein kinase 10	7	3.868	0.5525	1.1958
**1HOV**	72 kDa type IV collagenase	7	3.54	0.5057	1.19005
**19GS**	Glutathione S-transferase P	5	3.356	0.6711	1.1893
**1WMA**	Carbonyl reductase [NADPH] 1	5	3.295	0.659	1.18839
**1T46**	Mast/stem cell growth factor receptor	9	3.801	0.4223	1.18586
**2R7B**	3-phosphoinositide-dependent protein kinase 1	5	3.25	0.6501	1.17651
**1TJJ**	Ganglioside GM2 activator	6	3.354	0.559	1.17648
**1XDC**	Superoxide dismutase [Mn], mitochondrial	5	3.321	0.6643	1.17072
**3BYU**	Proto-oncogene tyrosine-protein kinase LCK	10	3.637	0.3637	1.15186

PDB ID	Target Name	Number of Features	Fit Score	Normalized Fit Score	z'-score
**3DZ4**	S-adenosylmethionine decarboxylase proenzyme	5	3.216	0.6432	1.12778
**1NMS**	Caspase-3	10	3.864	0.3864	1.11274
**2BXV**	Dihydroorotate dehydrogenase, mitochondrial	7	3.622	0.5174	1.10454
**1R1I**	Neprilysin	8	3.619	0.4524	1.08531
**1MA0**	Alcohol dehydrogenase class-3	6	3.725	0.6208	1.07455
**3CBS**	Cellular retinoic acid-binding protein 2	9	4.255	0.4727	1.06027
**1OSH**	Bile acid receptor	5	3.384	0.6769	1.04748
